# Characterization of High-Gamma Activity in Electrocorticographic Signals

**DOI:** 10.3389/fnins.2023.1206120

**Published:** 2023-08-07

**Authors:** Johannes Gruenwald, Sebastian Sieghartsleitner, Christoph Kapeller, Josef Scharinger, Kyousuke Kamada, Peter Brunner, Christoph Guger

**Affiliations:** ^1^g.tec medical engineering GmbH, Schiedlberg, Austria; ^2^Institute of Computational Perception, Johannes Kepler University, Linz, Austria; ^3^Department for Neurosurgery, Asahikawa Medical University, Asahikawa, Japan; ^4^Hokashin Group Megumino Hospital, Sapporo, Japan; ^5^National Center for Adaptive Neurotechnologies, Albany, NY, United States; ^6^Department of Neurosurgery, Washington University School of Medicine, St. Louis, MO, United States

**Keywords:** high-gamma activity, electrocorticography, brain-computer interface, high-gamma frequency band, biosignal processing

## Abstract

**Introduction:**

Electrocorticographic (ECoG) high-gamma activity (HGA) is a widely recognized and robust neural correlate of cognition and behavior. However, fundamental signal properties of HGA, such as the high-gamma frequency band or temporal dynamics of HGA, have never been systematically characterized. As a result, HGA estimators are often poorly adjusted, such that they miss valuable physiological information.

**Methods:**

To address these issues, we conducted a thorough qualitative and quantitative characterization of HGA in ECoG signals. Our study is based on ECoG signals recorded from 18 epilepsy patients while performing motor control, listening, and visual perception tasks. In this study, we first categorize HGA into HGA types based on the cognitive/behavioral task. For each HGA type, we then systematically quantify three fundamental signal properties of HGA: the high-gamma frequency band, the HGA bandwidth, and the temporal dynamics of HGA.

**Results:**

The high-gamma frequency band strongly varies across subjects and across cognitive/behavioral tasks. In addition, HGA time courses have lowpass character, with transients limited to 10 Hz. The task-related rise time and duration of these HGA time courses depend on the individual subject and cognitive/behavioral task. Task-related HGA amplitudes are comparable across the investigated tasks.

**Discussion:**

This study is of high practical relevance because it provides a systematic basis for optimizing experiment design, ECoG acquisition and processing, and HGA estimation. Our results reveal previously unknown characteristics of HGA, the physiological principles of which need to be investigated in further studies.

## 1. Introduction

The human brain's electrophysiology has been studied extensively over the past decades, dating back to the first electroencephalographic (EEG) recordings performed by Berger (1929). Since then, brain signals have been categorized into distinct frequency bands (e.g., delta, theta, alpha, beta, gamma, and high-gamma). Signals in these bands are commonly associated with various cortical processes that reflect different states of mind (Pfurtscheller and Lopes da Silva, 1999; Pfurtscheller, 2001). While non-invasive EEG can record brain signals below 50 Hz, observing the high-gamma frequency band (i.e., above ≈50 Hz) requires invasive recording techniques such as electrocorticography (ECoG) or stereo electroencephalography (sEEG).

**Figure 1:**
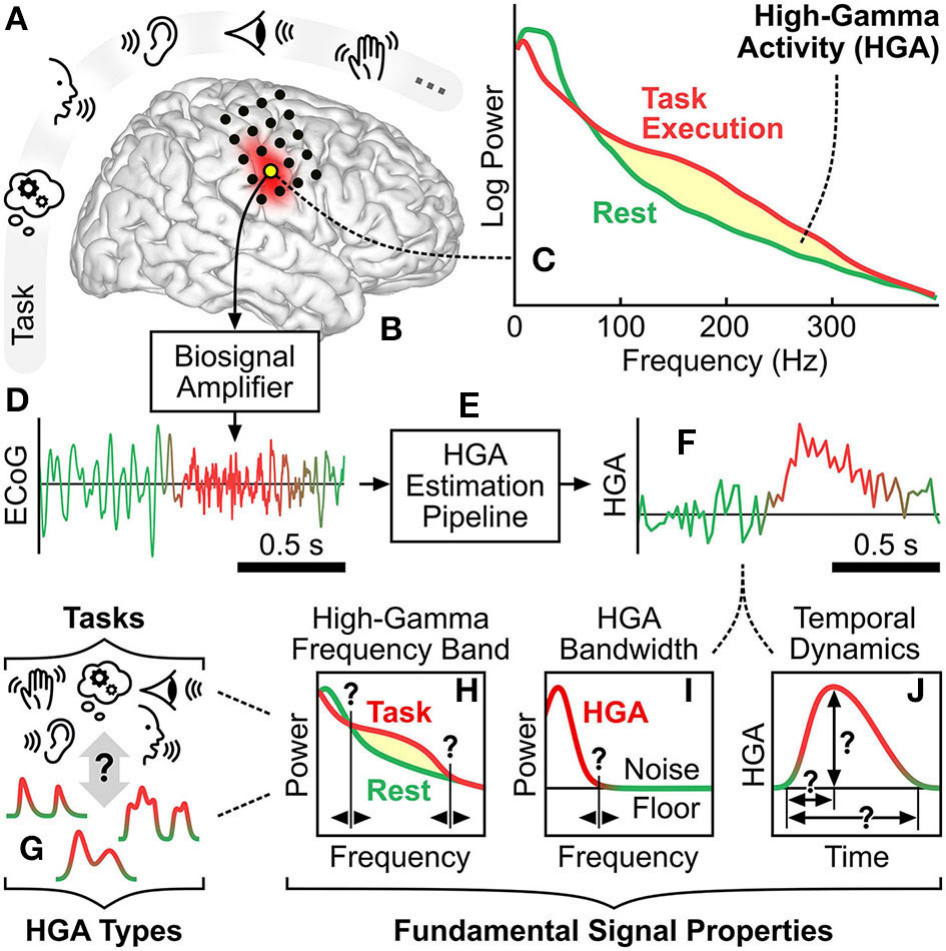
High-gamma activity: context and principles. **(A)** Exemplary cognitive and behavioral tasks involving memory, language, auditory, visual, and motor function. **(B)** Cortical region corresponding to such a cognitive or behavioral task. Black dots illustrate exemplary locations of implanted ECoG electrodes. **(C)** Typical task-related spectral change of a brain signal recorded at such a cortical region. Yellow shading illustrates the power increase due to HGA. **(D)** Typical time course of an ECoG signal acquired by a biosignal amplifier from a cortical region involved in the task. **(E)** Signal processing pipeline that estimates HGA from ECoG recordings. **(F)** Typical time course of estimated HGA. **(G)** HGA types associated with cognitive and behavioral tasks. **(H–J)** Illustration of the fundamental signal properties, i.e., the high-gamma frequency band, the HGA bandwidth, and the temporal dynamics of HGA.

Changes in high-gamma band power are commonly referred to as high-gamma activity (HGA). Over the past two decades, many studies have identified and confirmed HGA as a robust neural correlate of cognition and behavior. These studies encompass motor (Crone et al., 1998; Leuthardt et al., 2004; Canolty et al., 2006; Miller et al., 2007, 2010; Li et al., 2017; Wu et al., 2017; Pan et al., 2018; Gruenwald et al., 2019; Thomas et al., 2019), somatosensory (Menon et al., 1996; Canolty et al., 2006; Genetti et al., 2015; Prueckl et al., 2015; Wahnoun et al., 2015), auditory (Crone et al., 2001; Ray et al., 2003; Towle et al., 2008; Gaona et al., 2011; Potes et al., 2012; Sturm et al., 2014; Tamura et al., 2016), language (Leuthardt et al., 2004; Sinai et al., 2005; Towle et al., 2008; Pei et al., 2011a,b; Arya et al., 2017, 2018, 2019; Kambara et al., 2018; Williams Roberson et al., 2020), and visual (Ramot et al., 2012; Matsuzaki et al., 2013; Boucher et al., 2015; Miller et al., 2016; Rupp et al., 2017; Schalk et al., 2017; Kapeller et al., 2018a,b; Nakai et al., 2018; Wittevrongel et al., 2020) functions, as well as various types of cognitive (e.g., memory) tasks (Sederberg et al., 2003; Canolty et al., 2006; Axmacher et al., 2007; Lachaux et al., 2007; Ray et al., 2008; Burke et al., 2014; Kunii et al., 2014; Serruya et al., 2014; Noy et al., 2015; Ueda et al., 2015). Further studies demonstrated the role of HGA during resting state and sleep (Hirai et al., 1999; Freeman et al., 2000; He et al., 2008; Geller et al., 2014). It is important to note that HGA corresponds to a broadband power change and should not be confused with narrowband phenomena such as gamma oscillations (30–80 Hz; Hudson and Jones, 2022) or high-frequency oscillations (100–200 Hz; Staba et al., 2002), which potentially occur in the high-gamma frequency band. Figures 1A–F illustrate the context, principles, and typical estimation procedure of HGA.

HGA tracks cognitive and behavioral task engagement with high spatiotemporal fidelity and exhibits outstanding consistency over task repetitions (Miller et al., 2007). These qualities make HGA highly suitable for invasive brain-computer interface (BCI) applications, such as motor rehabilitation that provide prosthetic limb control and movement restoration (Leuthardt et al., 2004; Shenoy et al., 2008; Kubanek et al., 2009; Yanagisawa et al., 2011; Pistohl et al., 2012; Jiang et al., 2017; Li et al., 2017; Pan et al., 2018; Gruenwald et al., 2019; Thomas et al., 2019), speech prostheses that synthesize speech directly from the cortex (Leuthardt et al., 2004; Pei et al., 2011a; Herff et al., 2015), and decoding of visual perception (Rupp et al., 2017; Kapeller et al., 2018a,b). For all these applications to perform well, HGA extracted from ECoG must match the true physiological activity as closely as possible. This requires isolating the true physiological activity generated by the cognitive or behavioral task of interest from other physiological activity and the noise introduced by the HGA estimator. Common performance metrics for this context are Pearson's correlation coefficient, signal-to-noise ratio (SNR), the mean squared error (MSE), mutual information, etc.

The ability to precisely and robustly locate the cortical areas involved in cognitive and behavioral tasks from HGA has given rise to functional mapping applications that are now widely used in the presurgical evaluation of epilepsy and tumor patients. In these functional mapping applications, HGA identifies sensorimotor regions (Crone et al., 1998; Sinai et al., 2005; Leuthardt et al., 2007; Brunner et al., 2009; Hermes et al., 2010; Ruescher et al., 2013; Genetti et al., 2015; Prueckl et al., 2015; Wahnoun et al., 2015; Wu et al., 2017), expressive language regions and the auditory cortex (Sinai et al., 2005; Towle et al., 2008; Edwards et al., 2010; Roland et al., 2010; Pei et al., 2011b; Babajani-Feremi et al., 2016; Arya et al., 2017, 2018, 2019; Kambara et al., 2018), visual regions (Matsuzaki et al., 2013; Kapeller et al., 2018b; Nakai et al., 2018; Wittevrongel et al., 2020), and memory regions (Axmacher et al., 2007; Burke et al., 2014; Kunii et al., 2014). This identification of cortical regions is commonly realized by a statistical test that compares task-related changes in HGA to a resting-state condition. The sensitivity of such a statistical test critically depends on minimizing the noise introduced by the HGA estimator. Common performance metrics in this context are *z*-scores and the coefficient of determination (*r*^2^).

There exist a variety of qualitative and quantitative characteristics of HGA. One such qualitative characteristic is that different cognitive and behavioral tasks can produce different types of HGA. For example, a motor control task may produce a smooth HGA type in sensorimotor cortex with relatively slow transients, whereas a receptive or expressive language task may produce a burst HGA type in Broca's or Wernicke's area with relatively fast transients. Figure 1G illustrates this conceptual relationship between HGA types and cognitive and behavioral tasks. Figures 1H–J further illustrate three quantitative characteristics of HGA, which we also refer to as fundamental signal properties hereafter: First, the high-gamma frequency band is the set of adjacent spectral components subject to physiological task-related power modulation. The high-gamma frequency is typically defined by a lower and an upper cutoff frequency, e.g., 60–300 Hz. Second, the HGA bandwidth refers to the highest frequency component present in the HGA time course (e.g., 20 Hz). Third, the temporal dynamics of HGA describe the shape of task-related HGA time courses, e.g., in terms of rise time, duration, and amplitude. These three fundamental signal properties are likely to be different for each HGA type. We refer to the identification and assessment of qualitative and quantitative characteristics of HGA as HGA characterization hereafter.

Despite its extensive use in various application contexts, HGA has never been systematically characterized. However, such characterization is essential for various practical reasons. First, knowledge of the high-gamma frequency band is required to adjust fundamental recording and processing parameters (e.g., sampling rate of the biosignal amplifier; frequency band of the HGA estimator). Furthermore, knowledge of the HGA bandwidth is required to adjust the HGA estimator's feature rate (i.e., the number of HGA estimates computed per second) according to the sampling theorem. Finally, knowledge of the temporal dynamics of HGA is essential for experimental protocol design (e.g., with appropriate task duration) and for adjusting processing algorithms (e.g., with appropriate size and location of a BCI classifier window).

In this paper, we address the issues described above and present a systematic characterization of HGA in ECoG signals. This characterization is based on ECoG signals recorded from 18 epilepsy patients with temporarily implanted ECoG electrodes while they perform motor, listening, and visual perception tasks. In this study, we first categorize HGA into HGA types associated with cognitive/behavioral tasks. For each HGA type, we then systematically quantify the three fundamental signal properties of HGA identified above: (1) the high-gamma frequency band, (2) the HGA bandwidth, and (3) the temporal dynamics of HGA. In a final step, we summarize and discuss our results, focusing on their relevance to HGA estimation.

## 2. Materials and methods

### 2.1. Subjects

We evaluated ECoG signals recorded from 18 patients (S01–S18) with intractable epilepsy who underwent clinically indicated localization and subsequent resection of their seizure onset zone. For this purpose, the patients were implanted with subdural electrode grids and strips over their left and/or right hemispheres. The grids remained implanted for a duration of up to two weeks and were used for ECoG-based functional mapping to assist in surgical planning. S01–S11 were patients at Albany Medical College (Albany, New York), and S12–S18 were patients at Asahikawa Medical University (Asahikawa, Japan). All subjects in this study voluntarily participated in the research experiments, and written informed consent was obtained from each patient before participating in the study. The study was approved by the Institutional Review Boards of both Albany Medical College and Asahikawa Medical University. Table 1 summarizes subject demographics, electrode coverages, and performed experimental protocols. The individual electrode coverages for subjects S01–S18 are provided in Supplementary material (Section 1).

**Table 1:** Participating subjects, electrode coverages, and experimental protocols.

**ID**	**Age**	**Sex**	**Covered hemisphere**	**Electrodes total**	**Electrodes selected^a^**	**Performed Task(s)^b^**	**No. of Trials^a^**	**Amount of Data(s)^c^**
S01	36	Female	Right	112	30	L	4	80
S02	29	Female	Left	96	34	L	4	80
S03	25	Male	Right	112	24	L	4	80
S04	45	Male	Left	64	22	L	4	80
S05	49	Female	Left	80	29	L	4	80
S06	29	Female	Left	128	45	L	4	80
S07	25	Female	Left	128	65	L	4	80
S08	22	Male	Right	96	19	L	4	80
S09	34	Male	Left	64	22	L	4	80
S10	28	Male	Left	134	50	L	4	80
S11	27	Female	Left	98	45	L	4	80
S12	35	Female	Right	98	20	MC	90	270
S13	26	Male	Right	140	60	MC	120	360
S14	26	Male	Both	186	40	VP	140	140
S15	17	Female	Left	164	60 / 24	MC/VP	60/280	180 / 280
S16	22	Male	Right	158	60 / 40	MC/VP	240/280	720 / 280
S17	23	Male	Right	158	40	VP	280	280
S18	37	Male	Right	100	18 / 24	MC/VP	60/280	180 / 280

a If two numbers are given, they correspond to the respective protocol.

b MC, motor control; L, language; VP, visual perception (see Section 2.2.).

c Total length of collected data in seconds (rounded to integer values).

### 2.2. Cognitive and behavioral tasks

The subjects in this study performed three cognitive and behavioral tasks (see Table 1). These tasks were executed repeatedly and interleaved by a resting-state baseline interval. We refer to each of these repetitive executions as trials. To avoid subject fatigue, we split the experiments into several runs of manageable duration, where the subject performed a fixed number of trials (e.g., 20) without interruption.

#### 2.2.1. Motor control task

In this task, the subjects were visually cued to use their hand contra-lateral to the ECoG implant to perform a series of gestures from the well-known *rock-paper-scissors* hand game. We first verified that all subjects were able to perform the three gestures from this game. A screen placed approximately one meter in front of the subject visually cued the subjects to perform the gestures. For each trial in this experiment, the subject performed one gesture. A pictogram of one of the three different gestures was randomly shown for a duration of one second. Each cue was followed by a scrambled picture that served as a 1.5–2.5 s baseline interval. The subjects were instructed to form and hold the requested hand gesture on presentation of the corresponding cue, and to return to a relaxed position on presentation of the scrambled picture. One experimental run consisted of 20–30 trials per gesture (i.e., 60–90 in total). The sequence of gestures was randomized. In total, we collected 1–4 runs comprising a total of 60–240 trials per subject.

#### 2.2.2. Listening task

In this task, the subjects listened to four short narratives presented in their native language through loudspeakers placed in front of them. Before the narrative started, we recorded a baseline period where the subject was at rest and not exposed to any auditory input. To suppress environmental noise, we kept the room noise and distraction-free throughout the experiment. Each baseline interval and each narrative lasted for 10 s.

#### 2.2.3. Visual perception task

In this task, we presented the subjects with a battery of visual stimuli using a screen placed ≈1 m in front of them. At this distance, the stimuli spanned ≈12° (horizontally and vertically) of the visual field. Subjects were asked to keep fixated on the center of the screen. The visual battery comprised seven different categories (body parts, faces, digits, Hiragana words, Kanji words, line drawings, and simple objects), presented in a random sequence and shown in color or monochrome. Each visual stimulus appeared for 200 ms on the screen, followed by a black screen for a duration of 800 ms. Further details are provided in the original research paper (Kapeller et al., 2018b). One experimental run consisted of 40 trials per stimulus category (280 in total). We performed 1–2 runs for each subject.

### 2.3. Signal acquisition and preprocessing

We recorded ECoG signals sampled at 1.2 or 2.4 kHz using a g.HIamp biosignal amplifier (g.tec medical engineering GmbH, Austria) and processed the data in MATLAB (The Mathworks, Inc., Massachusetts, USA) using the g.HIsys High-Speed Online Processing for Simulink toolbox and g.BSanalyze (both g.tec medical engineering GmbH), or the general-purpose BCI2000 software platform (Schalk et al., 2004; Schalk and Mellinger, 2010). In total, we recorded signals from 2116 electrodes. We visually inspected these signals and discarded 148 electrodes affected by excessive noise or pathologic activity like epileptic discharges. From the remaining 1968 electrodes, we further narrowed down our selection to cortical areas known to be involved the corresponding cognitive or behavioral task. For example, we selected electrodes from sensorimotor areas for the motor control task. This finally yielded 771 electrodes across subjects S01–S18 selected for further processing (see Table 1).

To improve the signal quality, we applied a common average reference followed by notch filters at the line frequency and its harmonics (i.e., up to half the sampling frequency; Butterworth of order 6; cutoff frequencies at ±2.5 Hz at the respective center frequency). We further used a high-pass filter to remove low-frequency drifts from our recordings (first-order Butterworth; cutoff frequency at 5 Hz). These steps yielded our preprocessed ECoG signals.

### 2.4. HGA estimation

**Figure 2:**
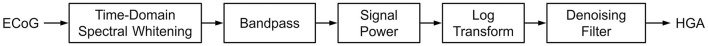
HGA estimation pipeline.

Figure 2 shows the HGA estimation pipeline used in this study, which is based on log band power extraction in time domain. This pipeline receives the preprocessed ECoG signals (see Section 2.3) as input. First, a time-domain spectral whitening filter (inverse autoregressive filter of order 10; see Gruenwald et al., 2019 for details) is applied. This step balances the power-law ECoG spectrum of the input signal so that all frequency components equally contribute to the subsequently computed band power. Second, a bandpass filter is applied (Butterworth of order 10), which removes all signal components outside the specified lower and upper cutoff frequencies. Third, the signal power is extracted as the mean squares over consecutive, non-overlapping windows of 10 ms length. This step produces HGA estimates at a rate of 100 Hz. Fourth, a log transform is applied, which (1) converts the asymmetric (e.g., χ^2^) distribution of the HGA estimation noise to a more Gaussian distribution and (2) decouples the variance of the estimation noise from the signal mean, leading to favorable stationary conditions (Bartlett and Kendall, 1946). Finally, an optional Butterworth lowpass filter (order 6; cutoff frequency 10 Hz) is applied to denoise the HGA estimates. Some parameters of this pipeline (e.g., bandpass cutoff frequencies) change during our analyses. We provide concrete values when they are available.

### 2.5. HGA characterization

This section describes our HGA characterization analyses. In a first qualitative step, we identified individual HGA types (Section 2.5.1). Based on the identified HGA types, Sections 2.5.2–2.5.4 present our quantitative characterization of the fundamental signal properties of HGA, i.e., the high-gamma frequency band, the HGA bandwidth, and the temporal dynamics of HGA.

#### 2.5.1. Identifying HGA types

According to our experience and HGA reported in the literature, we identified three common HGA types associated with cognitive and behavioral tasks: (1) Smooth HGA is characterized by a smooth activation pattern and relatively slow transients. This type of HGA can be found within sensorimotor cortex in motor control experiments. (2) Burst HGA is characterized by burst activation and fast to intermediate transients. This temporal activation pattern can be found within Broca's area, Wernicke's area, and the auditory cortex during receptive or expressive language tasks. (3) Pulsed HGA exhibits short pulses with fast transients and is produced by the visual cortex and the fusiform gyrus in response to visual stimuli. Note that the cognitive and behavioral tasks considered in this study (i.e., motor control, listening, and visual perception; see Section 2.2) correspond to these HGA types.

#### 2.5.2. High-gamma frequency band

Determining the high-gamma frequency band requires finding a pair of lower and upper cutoff frequencies within which the proportion of physiological, task-related power modulation in HGA estimates reaches a maximum. To solve this maximization problem, we performed a grid search across lower and upper cutoff frequencies, where we used *z*-scores as the output metric.

Our grid search comprised 15 logarithmically spaced values between 30 and 100 Hz for the lower cutoff frequency and 10 logarithmically spaced values between 110 and 500 Hz for the upper cutoff frequency, where we excluded all pairs of lower and upper cutoff frequencies yielding a bandwidth of < 30 Hz (e.g., 100–110 Hz). For each of the remaining pairs, we first computed the HGA estimates using the pipeline described in Section 2.4 (without the denoising filter to preserve a maximum of statistical independence). Second, we epoched the HGA estimates into trials, based on task onsets stored in each recording file alongside the ECoG signals. The task-specific duration of these trials encompassed a pre-onset resting-state interval and a post-onset task activity interval. For the motor control, language, and visual perception task, we set the pre-onset interval to 0.75, 10, and 0.25 s, and the post-onset interval to 1.5, 10, and 0.5 s, respectively. Third, we offset-corrected each trial by subtracting the mean HGA during the pre-onset interval. Fourth, we computed one *z*-score defined as the mean HGA increase Δμ from the pre-onset interval to the post-onset interval (averaged across trials), normalized by the standard deviation σ_pre_ of all samples from all trials within the pre-onset interval:

(1) $$\begin{array}{l} z=\Delta \mu /\sigma_{\text{pre}}. \end{array}$$

This procedure yielded a 15 × 10 (lower cutoff × upper cutoff frequency) heatmap of *z*-scores for all electrode channels, subjects, and tasks. In a fifth step, we then combined the electrode channels as a weighted average into one subject-specific *z*-score heatmap per task, where the weights corresponded to the maximum *z*-score of the respective electrode channel.

#### 2.5.3. HGA bandwidth

The HGA bandwidth refers to the highest frequency component present in the HGA time courses. To compute these HGA time courses, we used the HGA estimation pipeline shown in Figure 2 (without denoising filter), where we adjusted the cutoff frequencies of the bandpass filter for each cognitive and behavioral task individually, based on the previously obtained results from the high-gamma frequency band characterization step (see Section 2.5.2 and **Figure 4**). Specifically, we used 70–300, 50–140, and 50–200 Hz for the motor control task, listening task, and visual perception task, respectively.

We then employed the recently published SNR decomposition method to extract the HGA bandwidth from these HGA estimates (Gruenwald et al., 2021). The SNR decomposition method allows the unsupervised quantification of underlying physiological activity in noisy HGA estimates. Here, the term *unsupervised* means that the SNR decomposition method does not require any task-related information about the cognitive or behavioral task, which makes this method universally applicable to ECoG signals.

**Figure 3:**
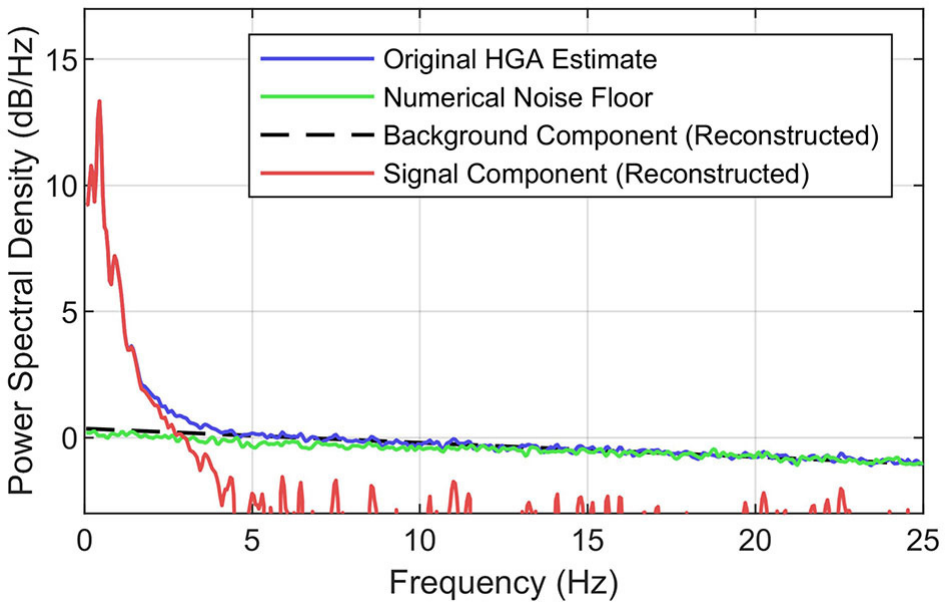
Concept of the SNR decomposition method. Illustration of decomposing the PSD of the original HGA estimate (blue) into the background component (black, dashed) and the signal component (red). The numerical noise floor obtained via synthesized ECoG is shown in green. Exemplary data set from the motor control task.

Figure 3 illustrates the principles of the SNR decomposition method, which separates the PSD of HGA estimates *P*_*x*_(*f*) into a signal component *P*_*s*_(*f*) dominant at lower frequencies and a background component *P*_*w*_(*f*) dominant at higher frequencies, i.e., *P*_*x*_(*f*) = *P*_*s*_(*f*)+*P*_*w*_(*f*), where *f* denotes the frequency. This power-domain additivity directly follows from the fact that log band power based HGA estimates *x*[*n*] are the sum of an underlying, task-related signal component *s*[*n*] and a stationary background component *w*[*n*], i.e., *x*[*n*] = *s*[*n*]+*w*[*n*] (Gruenwald et al., 2017), where *n* denotes the discrete-time index. This background component *w*[*n*] comprises the numerical estimation noise floor but might also include a physiological HGA component that is unrelated to the task.

To better understand this background component, we investigated the numerical estimation noise floor. For this purpose, we used a simple synthesis technique: first, we computed an autoregressive model (order 20) of the preprocessed ECoG signals recorded from each electrode for a given data set. Second, we generated random noise with the same length and dimensionality as the recorded ECoG and used the autoregressive models to produce signals with exactly the same spectral characteristics as the recorded ECoG but without HGA. Third, we applied the HGA estimator (see Figure 2) to this synthesized data set. Fourth, we calculated the PSD of the estimator output, which yielded the desired numerical noise floor. Figure 3 shows this numerical noise floor, which decreases linearly toward higher frequencies. This is because HGA estimation noise is slightly serially correlated since HGA estimates are calculated from a bandpass signal that is also serially correlated. Figure 3 also shows that the numerical noise floor dominates the background component, as the gap between the PSD of the HGA estimates and the numerical noise floor almost vanishes above 5 Hz.

To obtain the background component based on all these observations, the SNR decomposition method fits a straight line into the linear regime of the original PSD, e.g., above 5 Hz. The signal component is then obtained by subtracting the background component from the original PSD in the linear domain. Given this decomposition, the HGA bandwidth then corresponds to the frequency where the signal PSD falls below a certain threshold relative to the background component. We chose −3 dB (half noise power) as the threshold, which is a common value in experimental signal power analysis.

To keep our analysis tractable, we extracted one HGA bandwidth specific to each subject and task. For this purpose, we first averaged the original PSDs over all channels for each subject and task. To make our approach more robust, we then smoothed the resulting subject-specific original PSDs via a symmetric moving-average filter of 20 samples corresponding to a frequency resolution of 0.025 Hz. Finally, we extracted the HGA bandwidth from these smoothed, subject-specific original PSDs as described above.

#### 2.5.4. Temporal dynamics of HGA

The third and last fundamental signal property is a set of parameters that describe the temporal dynamics of HGA. As temporal dynamics of HGA we consider the task-related (1) rise time, (2) duration, and (3) amplitude of HGA. Our method automatically identified onsets of task-related HGA and created trials based on them. We then extracted the temporal dynamics for each of these trials and generated a statistical representation across cognitive and behavioral tasks, following the procedure described below.

(1) For each subject and task, we pre-selected five channels with strongest task-related HGA in a preliminary mapping analysis.
(2) We computed HGA estimates for these pre-selected channels employing the pipeline shown in Figure 2, including the denoising filter and using the same task-specific bandpass cutoff frequencies as in Section 2.5.3.
(3) We offset-corrected the resulting HGA estimates by the mean value during resting-state. Standard approaches, e.g., based on the signal mean or median, were not appropriate here because these approaches are positively biased due to task-related HGA present in the signal. Instead, we implemented a more robust concept based on simple histogram analysis. We observed that the histogram of lowpass-filtered HGA estimates is composed of two components: first, a dominant stationary Gaussian component representing the estimation noise at the baseline level, and second, a non-stationary task-related component manifested by a pronounced right tail. Based on this composition, we determined the baseline level as the histogram peak location, i.e., the mean of the dominant stationary Gaussian component. This histogram peak location is not shifted by the right tail of the histogram, which makes this approach robust against a task-related bias. We offset-corrected all electrode channels by the respective resting-state level. Supplementary material (Section 2) illustrates this offset correction step.
  In the following notation, we omit any reference to electrode channels, subjects, tasks, or trials for convenience and conciseness.
(4) We computed the symmetric difference of *s*[*n*] to quantify its slope, i.e.,

(2) $$\begin{array}{l} d\left[n\right]=s\left[n+N_{s}/2\right]-s\left[n-N_{s}/2\right], \end{array}$$

where *N*_*s*_ = *T*_*s*_/*T* = 16 with *T*_*s*_ = 0.16 s as a robust HGA rise time average and the HGA estimation interval *T* = 0.01 s.
(5) We detected the onset of task-related HGA whenever *d*[*n*]>0.25 (threshold empirically determined) for at least *N*_*s*_ samples. Then, we epoched *s*[*n*] into trials based on the detected onsets (pre- and post-onset duration: 3.0 s and 5.5 s, respectively).
(6) For the motor control and listening task, we removed trials where no task or stimulus was present. We omitted this step for the visual perception task due to its high pace that made it difficult to differentiate between resting-state and activation periods.
(7) We processed each of the remaining trials as follows:
(7a) We determined the peak location *n*_pk_ and amplitude *s*_pk_ = *s*[*n*_pk_].
(7b) We removed all trials with peak amplitude *s*_pk_ < 1.0 (empirical threshold).
(7c) We located the beginning *n*_1_ of the task-related HGA as the last zero-crossing of *s*[*n*] before the peak *n*_pk_.
(7d) Likewise, we located the end *n*_2_ of the task-related HGA where *s*[*n*] first fell below zero after *n*_pk_.
(7e) Intuitively, we could compute the task-related rise time and duration directly from *n*_1_, *n*_pk_, and *n*_2_. However, this would yield inaccurate results because *n*_1_ and *n*_2_ were obtained via thresholding, which is prone to errors for noisy signals. To overcome this issue, we developed a robust approach to extract the rise time and duration based on the area under the curve (AUC). For this purpose, we express the AUC *A* of the complete trial as

(3) $$\begin{array}{ll} A & =T\overset{n_{2}-1}{\underset{k=n_{1}}{\sum }}s\left[k\right] \end{array}$$

(4) $$\begin{array}{l} =pT\left(n_{2}-n_{1}\right)s_{\text{pk}} \end{array}$$

(5) $$\begin{array}{l} =pT_{d}s_{\text{pk}}. \end{array}$$

In Equation 4, we substituted the sum by *p*(*n*_2_−*n*_1_)*s*_pk_, where 0 < *p* < 1 indicates how much of the bounding rectangle *T*(*n*_2_−*n*_1_) × *s*_pk_ (width × height) is filled by *A*. In a next step, we recognized that *T*(*n*_2_−*n*_1_) is equivalent to the duration, which we introduced as *T*_*d*_ and substituted accordingly in Equation 5. In a last step, we rewrote Equation 5 to compute *T*_*d*_ via.

(6) $$\begin{array}{l} T_{d}=\frac{A}{ps_{\text{pk}}}. \end{array}$$

While *A* and *s*_pk_ can be determined from Equation 3 and step (7a), respectively, *p* is unknown in general. Fortunately, *p*≈0.5 is a robust approximation in practice. This approximation is justified by the fact that the AUC begins filling the bounding rectangle *T*_*d*_×*s*_pk_ (width × height) from the lower left corner (*s*[*n*_1_] = 0) to the top (*s*_pk_ at *n*_pk_) and back to the lower right corner (*s*[*n*_2_] = 0). This corresponds to *p* = 0.5, i.e., an AUC that fills exactly 50% of the bounding rectangle. Consequently, we computed the duration via Equations 3 and 6, step (7a), and *p* = 0.5.
(7f) We computed the rise time analogously to the previous step, where we substituted *n*_2_ by *n*_pk_ in Equation 3 to obtain the corresponding AUC.
(8) We grouped the obtained temporal dynamic measures (i.e., rise time, duration, and amplitude) by task to create a statistical representation.

## 3. Results

**Figure 4:**
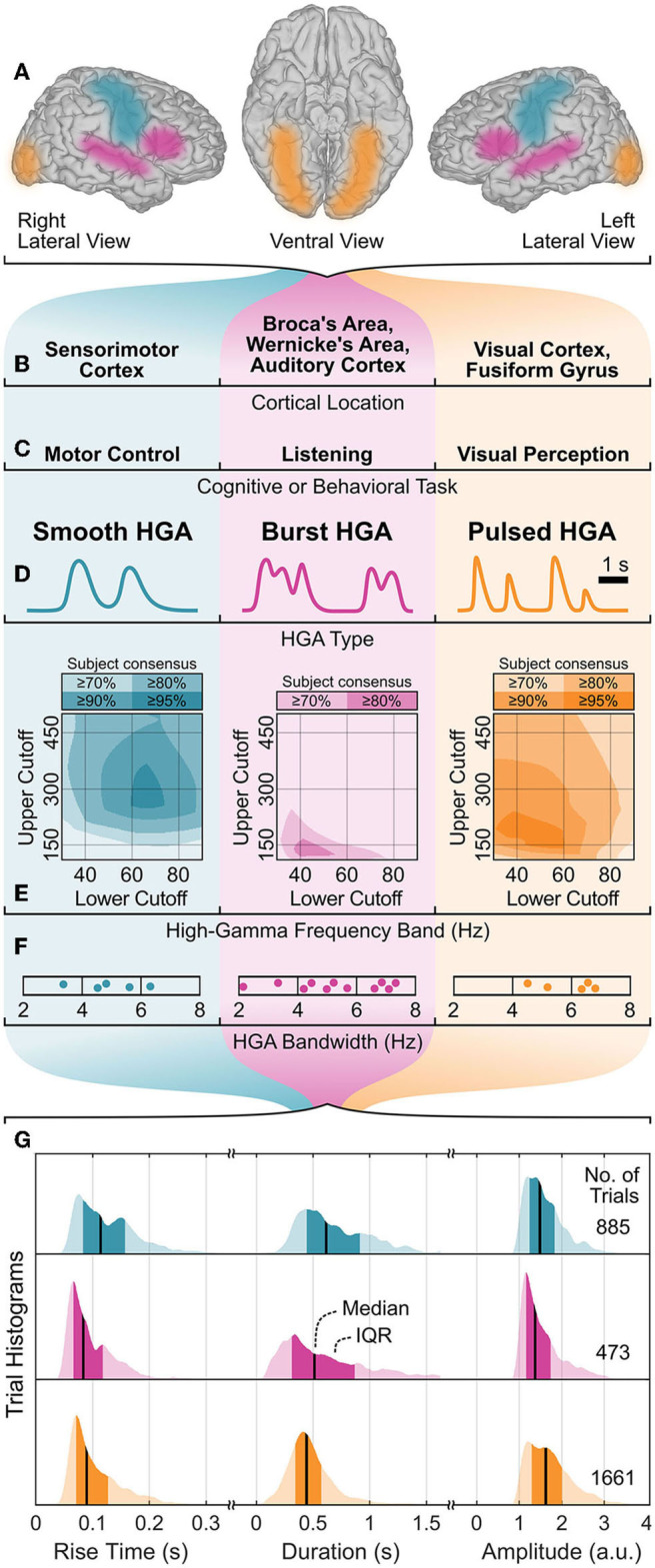
HGA characterization summary. **(A, B)** Cortical locations, **(C)** associated cognitive and behavioral tasks, and **(D)** exemplary time courses of HGA types with corresponding **(E)** high-gamma frequency band, **(F)** HGA bandwidth, and **(G)** temporal dynamics of HGA. Shaded areas **(E)** represent the subject consensus, indicating that all subjects exceed the corresponding threshold. Dots **(F)** represent individual subjects. IQR, interquartile range; a.u., arbitrary unit.

In the first qualitative HGA characterization step, we identified three HGA types as smooth, burst, and pulsed HGA. Figures 4A–D illustrate this categorization with corresponding cognitive and behavioral tasks, involved cortical locations, and HGA time course illustrations. Figures 4E–G also summarize the results of the quantitative HGA characterization, which are covered more thoroughly in Sections 3.1–3.3.

### 3.1. High-gamma frequency band

Figure 4E shows the high-gamma frequency bands as shaded overlays. These shaded overlays indicate that all subjects exceed the specific threshold (e.g., 80%) relative to their maximum *z*-score (subject consensus). Consequently, all pairs of lower and upper cutoff frequencies within the area of the highest subject consensus can be regarded as the high-gamma frequency band. For example, 70–300 Hz (95% subject consensus), 50–140 Hz (80% subject consensus), and 50–200 Hz (95% subject consensus) are appropriate high-gamma frequency bands for the motor control, listening, and visual perception task, respectively.

**Figure 5:**
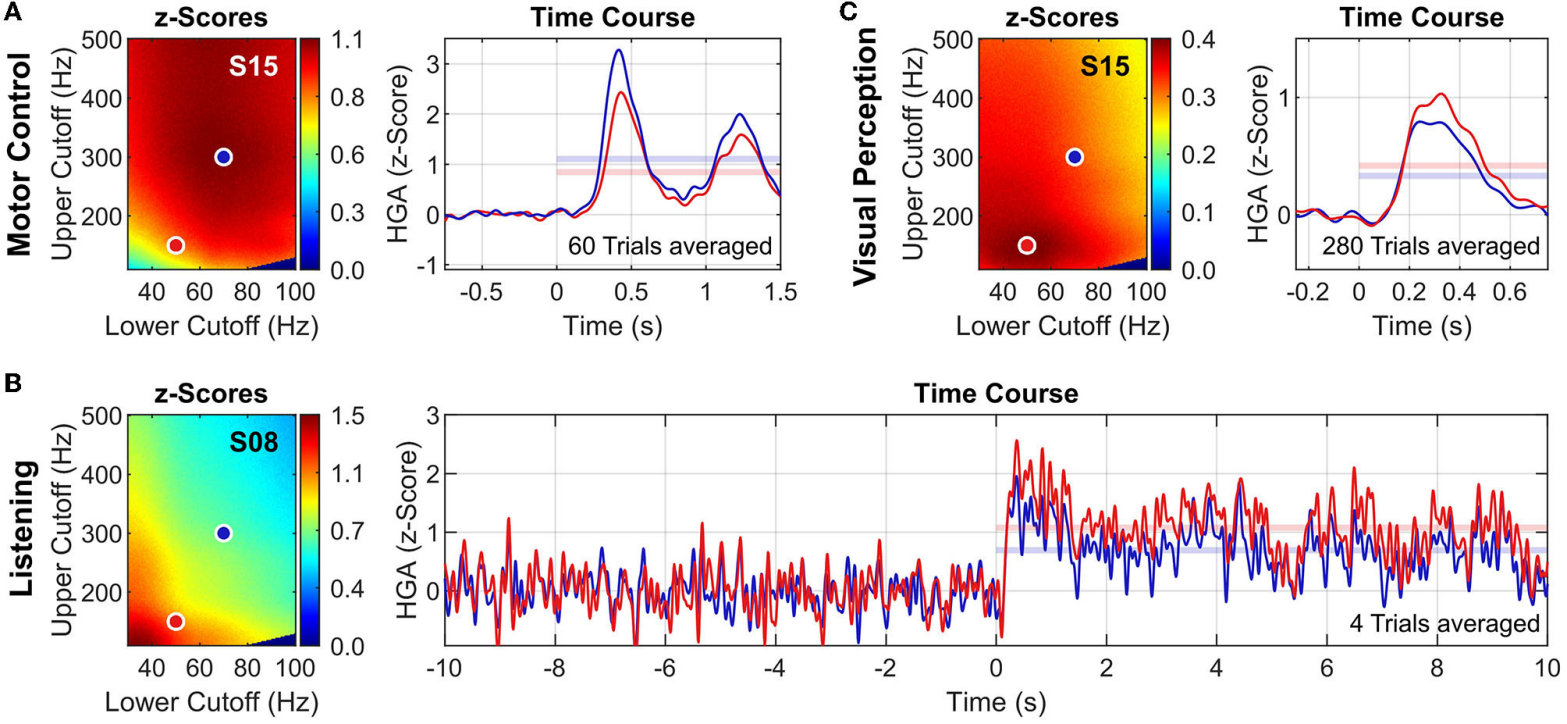
High-gamma frequency band analysis. Exemplary results for the motor control task **(A)**, the listening task **(B)**, and the visual perception task **(C)**. For each tasks, *z*-score heatmaps **(left)** and HGA time courses **(right)** of exemplary subjects and channels are shown. Two high-gamma frequency bands are illustrated: 50–150 Hz (red dots/traces) and 70–300 Hz (blue dots/traces). HGA time courses are presented as *z*-scores (averaged across all trials) with applied lowpass filter (Butterworth order 6, cutoff frequency 10 Hz, applied bidirectionally) to improve visualization. Translucent horizontal bars in the time course plots indicate the mean *z*-score during task activity, corresponding to the respective value in the *z*-score heatmap. Note that the noise of the HGA time courses depends on the number of averaged trials (see also Table 1).

Figure 5 presents exemplary results of the high-gamma frequency band analysis for each task. To underline the impact of the high-gamma frequency band on HGA estimation, we show the analysis results for two high-gamma frequency bands: 50–140 Hz (red dots/traces) and 70–300 Hz (blue dots/traces). Figures 5A, B both show results for S15 to illustrate task-related variations of the high-gamma frequency band within the same subject.

### 3.2. HGA bandwidth

**Figure 6:**
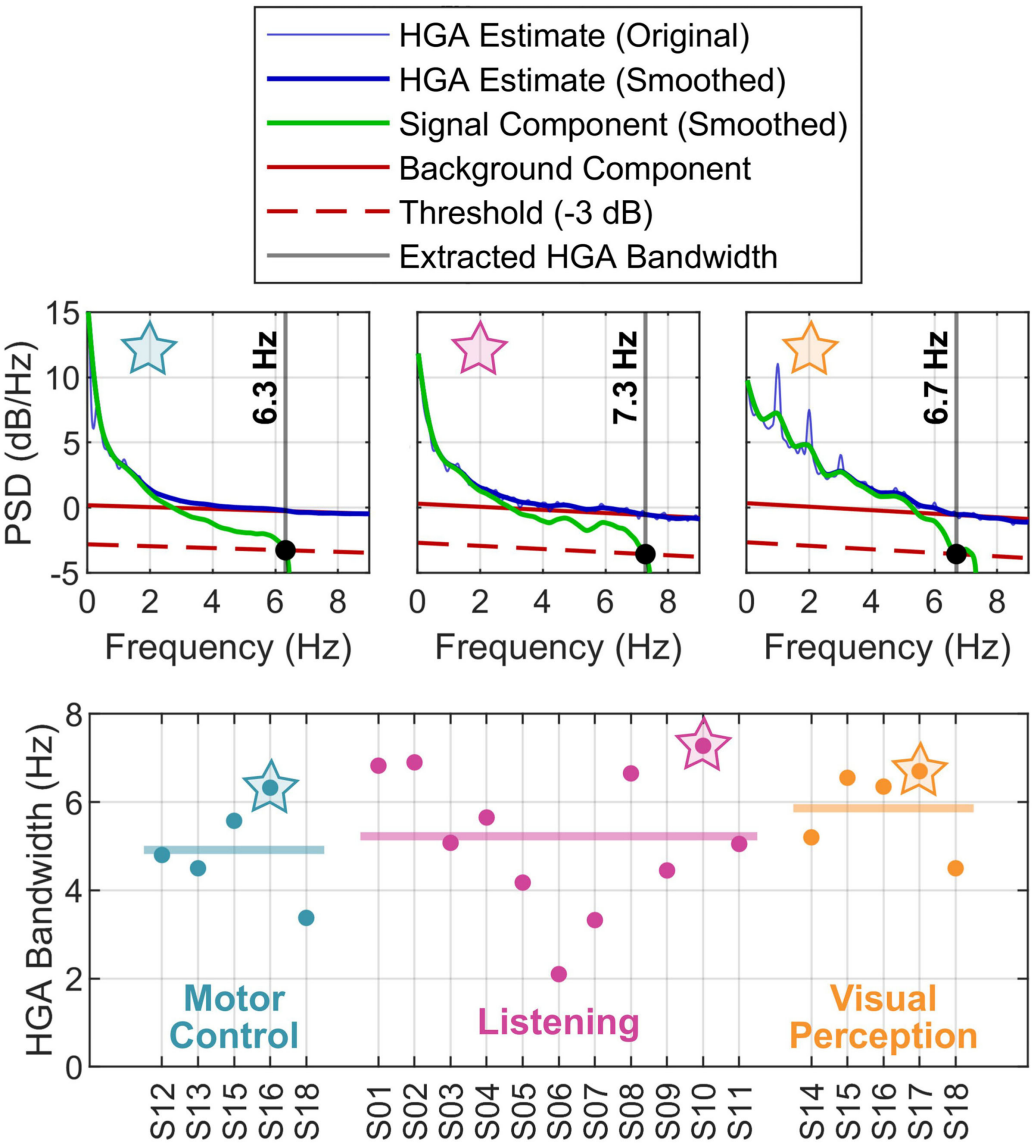
HGA bandwidth analysis. **(Top)** PSDs of exemplary subjects (motor control task: S16; listening task: S10; visual perception task: S17). **(Bottom)** Subject-specific HGA bandwidth, grouped by task. The thick horizontal lines indicate the mean across subjects. The star symbols relate top and bottom plots.

Figure 6 presents the results of the HGA bandwidth characterization. The PSD decomposition plots in the top row illustrate our concept for one exemplary subject per cognitive or behavioral task. The bottom row reports the HGA bandwidth of the individual subjects in each task. For the motor control task, the HGA bandwidth ranged from 3.4 to 6.3 Hz (4.9 Hz on average). For the language task, we obtained an HGA bandwidth from 3.2 to 6.8 Hz with 5.0 Hz on average. Finally, the HGA bandwidth in the visual perception task ranged from 4.3 to 6.5 Hz (5.8 Hz on average). All these results are also summarized in Figure 4F.

### 3.3. Temporal dynamics of HGA

**Figure 7:**
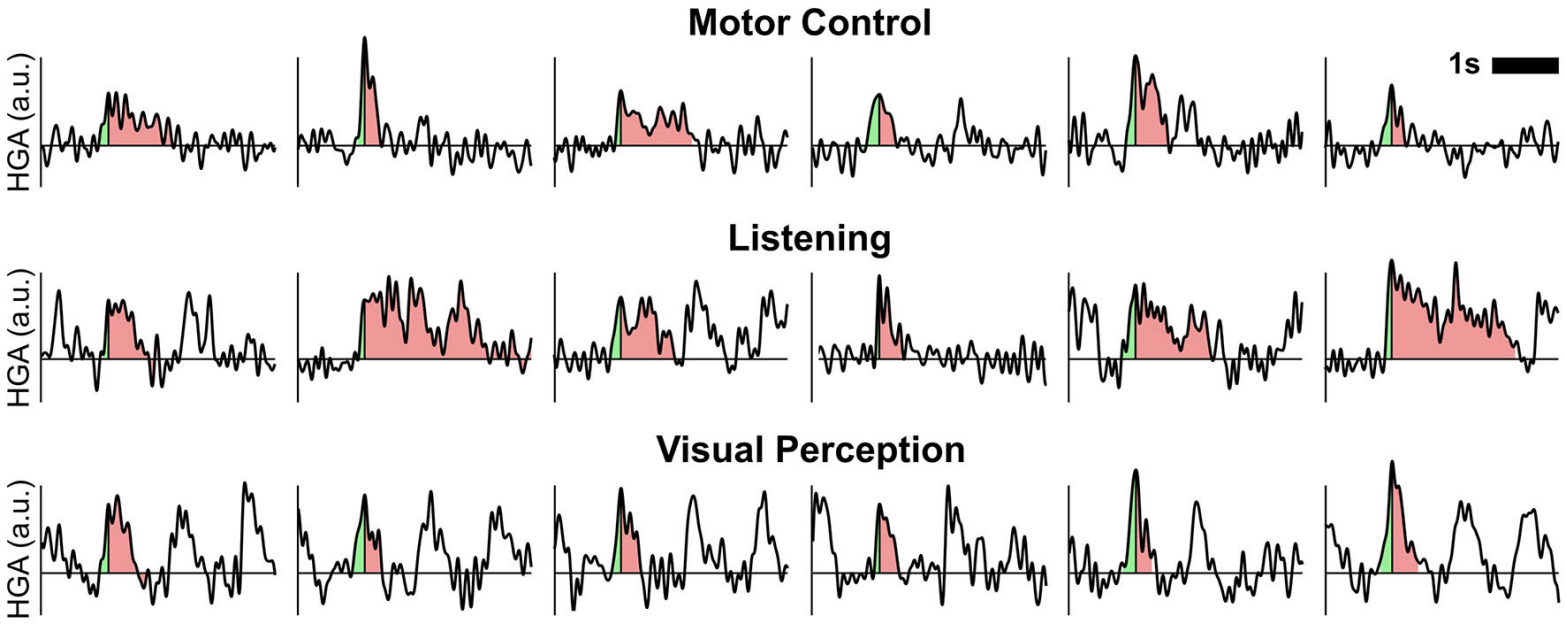
Exemplary time courses of detected HGA trials. Shaded areas relate to the rise time (light green) and decay (light red) of one trial. Combining the rise time and decay corresponds to the overall trial duration. a.u., arbitrary unit.

Figure 7 shows exemplary time courses of detected HGA trials for each cognitive or behavioral task. In these time courses, we indicated the rise time and the decay (i.e., from peak to end of trial) in green and red shading, respectively. Combining the rise time and the decay in these plots yields the overall duration.

Figure 4G summarizes the extracted temporal dynamics of HGA as trial histograms of the task-related rise time, duration, and amplitude extracted from real ECoG recordings. These histograms indicate each median and interquartile range (IQR), which we report as follows: For smooth, burst, and pulsed HGA, we obtained a respective median rise time of 114 (IQR: 83–157), 83 (66–118), and 90 (71–127) milliseconds, a median duration of 616 (444–913), 513 (313–868), and 444 (345–572) milliseconds, and a median amplitude of 1.49 (1.24–1.83), 1.37 (1.16–1.74), and 1.63 (1.30–2.01) arbitrary units.

## 4. Discussion

### 4.1. High-gamma frequency band

The high-gamma frequency band varies considerably across cognitive and behavioral tasks and between different subjects. Figure 4E shows that these variations across subjects can be moderate, such that a relatively wide range of upper and lower cutoff frequencies can be considered a subject-independent high-gamma frequency band for a specific task (high subject consensus). For example, 70–300 Hz yields 95% subject consensus for motor control and 50–200 Hz yields 95% for visual perception. For the listening task, the high-gamma frequency band varies greatly across subjects, so that only a small range in the vicinity of ≈50–140 Hz yields a rather low subject consensus of 80%.

To complicate things further, the high-gamma frequency band may even vary within the same subject depending on the cognitive or behavioral task. For example, S15 exhibited substantially different high-gamma frequency bands for the motor control and the visual perception tasks (see Figures 5A, B). To our knowledge, such systematic variations have not been reported before. Understanding and interpreting the neurophysiological principles governing these variations requires further experiments and analyses that are beyond the scope of this paper.

From a practical perspective, however, high-gamma frequency band characterization has two important implications. First, the upper cutoff of the high-gamma frequency band determines the minimum required ECoG recording sampling rate via the Nyquist-Shannon sampling theorem. For example, cognitive/behavioral tasks with an upper high-gamma frequency band cutoff frequency of 300 Hz (motor control, visual perception) require an ECoG recording sampling rate of at least 600 Hz. There is no point in using much higher sampling rates (e.g., 2.4 or 4.8 kHz), unless other phenomena at higher frequencies are of interest.

The second practical implication is that variations in the high-gamma frequency band must be addressed by the HGA estimation procedure, which is also underlined by the amplitude variations of the HGA time courses in Figure 5. Specifically, it is essential to customize the lower and upper cutoff frequencies of the HGA estimator for each subject and task to achieve optimal performance. This optimum can be found using our strategy presented in Section 2.5.2, i.e., by maximizing *z*-scores in a grid search across lower and upper cutoff frequencies. When task-specific information is not available in the data, our previously published SNR decomposition method can be employed for this maximization problem (Gruenwald et al., 2021). The SNR decomposition method allows quantifying (and thus maximizing) physiological, task-related HGA in ECoG signals without actual information about the experimental protocol.

It is important to note that the optimal lower and upper cutoff frequencies of an HGA estimator strongly depend on whether spectral whitening is enabled. This is intuitive because spectral whitening changes the frequency spectrum of the ECoG signal and thus alters the task-related contribution of each spectral component to the overall HGA estimate. Consequently, such a change in the frequency spectrum leads to different optimal lower and upper cutoff frequencies. If spectral whitening is disabled, for example, higher frequency components contribute much less to the overall HGA estimate due to the 1/*f* power-law ECoG spectrum (Miller et al., 2007, 2009). As a consequence, the high-gamma frequency bands identified in this study are only directly applicable to HGA estimators with spectral whitening enabled.

To overcome this limitation, we provide an analysis of the high-gamma frequency band using an HGA estimator without spectral whitening (see Section 3 in Supplementary material). This analysis yielded a high-gamma frequency band of 90–500 Hz (90% subject consensus) for motor control, 60–500 Hz (80% subject consensus) for listening, and 80–500 Hz (90% subject consensus) for visual perception.

### 4.2. HGA bandwidth

The HGA bandwidth varies greatly across subjects, implying that the transients of the HGA time courses are faster in some subjects than in others. This interpretation is supported by the relatively wide rise time histograms in Figure 4G. At first glance, we were surprised that the HGA bandwidth appears to be well below 10 Hz in all cases. Determining the relationship between the HGA bandwidth and the corresponding rise times allowed us to verify the plausibility of our results. This relationship is based on the assumption that the rise time *T*_*r*_ corresponds to the fastest possible ascent from minimum to maximum in a signal, which is approximately half the period *T* of the highest frequency component therein, i.e., *T*_*r*_≈*T*/2. This highest frequency component, in turn, approximately corresponds to the bandwidth *B* of the signal, so that *T*≈1/*B* and consequently *T*_*r*_≈1/2*B*. Substituting our experimentally determined HGA bandwidths into this equation, e.g., the total range of ≈3.2–6.5 Hz, yields corresponding rise times of about ≈70–150 ms, which corresponds approximately to the range of the determined rise times shown in Figure 4G.

Using the Nyquist-Shannon sampling theorem again, our HGA bandwidth characterization results can be formulated as an important rule of thumb for HGA estimation: Since HGA transients are band-limited by 10 Hz, HGA estimates computed at a rate of 20 Hz (cf. Nyquist rate) already cover all components of the underlying signal. While oversampling (i.e., using a multiple of the Nyquist rate for HGA estimation) may offer advantages for certain signal processing tasks and filters, higher HGA estimation rates do not capture additional information of the underlying, physiological source signal.

### 4.3. Temporal dynamics of HGA

The obtained temporal dynamics support the concept of categorizing HGA into different types corresponding to cognitive or behavioral tasks. Specifically, the rise times of smooth HGA (median: 114 ms; IQR: 83–157 ms) are considerably longer than those of burst HGA (83 ms; 66–118 ms) and pulsed HGA (90 ms; 71–127 ms). In addition, pulsed HGA has a consistently short trial duration (median: 444 ms; IQR: 345–572 ms), which contrasts with the relatively wide trial duration range of smooth HGA (616 ms; 444–913 ms) and burst HGA (513 ms; 313–868 ms). These rise times and durations are valuable information for designing experimental protocols. For example, our analyses have shown that the pace of the visual perception task (stimulus duration: 200 ms, one trial per second) was too fast for some subjects and therefore produced contaminated resting-state baseline segments. Interestingly, we did not observe significant amplitude differences between HGA types. This finding is important for functional mapping applications, e.g., for adjusting significance thresholds.

The range of our obtained rise times contradicts results from recent research, which reported much faster HGA transients (Coon and Schalk, 2016; Coon et al., 2016). We addressed this contradiction in an additional analysis provided in Supplementary material (Section 4). Surprisingly, the results of this analysis strongly suggest that the sharp HGA onset peaks produced by Coon and Schalk are noise artifacts. These findings should be addressed more thoroughly in future work.

### 4.4. Methodological consistency

It is essential to ensure that our findings are methodologically consistent. For this reason, we performed additional HGA characterizations with different HGA estimators and compared the results. In such an additional HGA characterization, for example, we disabled spectral whitening or used the Hilbert transform instead of log band power estimates.

Our results confirmed the strong impact of spectral whitening on the high-gamma frequency band analysis, which we already expected and discussed in Section 4.1. In addition, our analyses confirmed that the HGA bandwidth is well below 10 Hz, regardless of whether spectral whitening is enabled or the Hilbert transform is used. Finally, all considered HGA estimators yielded similar temporal dynamics of HGA (except for the amplitudes of the Hilbert transform, which are inherently larger since no log transform is used). We provide more details in Supplementary material (Section 3). Overall, these additional HGA characterization analyses yielded expected results, confirming the methodological consistency of our approach.

### 4.5. Limitations and remaining challenges

Our HGA characterization study includes three cognitive and behavioral tasks. To our knowledge, this is the most extensive experimental coverage ever considered in a single ECoG study; however, it is limited given the large number of tasks ever performed in ECoG experiments. A further limitation is that our study covers only one experimental protocol per task, and these protocols differ considerably. For this reason, we could not investigate the impact of the experimental protocol (e.g., stimulus type, duration, pace, intensity). However, such an investigation would have been beyond the scope of our study due to the enormously increased complexity.

For simplicity and clarity, we assumed a direct correspondence between cognitive and behavioral tasks and HGA type. Unfortunately, this relationship is ambiguous in reality. For example, we associated visual categorization tasks with pulsed HGA. However, pulsed HGA might be as well produced in a listening task (e.g., short words or auditory beeps) or a motor control task (e.g., by rapid and discontinuous hand movements). Similarly, processing a continuous stream of visual information is still a visual perception task, but might produce burst HGA or even smooth HGA. To resolve this ambiguity and avoid misconceptions, the results of our quantitative HGA characterization must be associated with *either* cognitive/behavioral tasks or HGA types. Therefore, we associate the high-gamma frequency band with cognitive/behavioral tasks rather than HGA types because the high-gamma frequency band is independent of the HGA time course (and, consequently, independent of the HGA type). In contrast, the HGA bandwidth and the temporal dynamics of HGA are characterized based on the HGA time course, so we associate these characteristics with HGA types rather than cognitive/behavioral tasks.

### 4.6. Outlook and further work

Further studies are needed to complement our findings. For example, it is important to better understand the mechanisms that govern the variation of the high-gamma frequency band and the HGA bandwidth across individual subjects in some cognitive or behavioral tasks. For example, it may be of interest to relate these results to subjects' behavioral or cognitive abilities and disabilities, e.g., intelligence quotient (IQ), reaction times, motor agility, cognitive diseases such as dementia, etc. Further studies should also address the variety of HGA types that can be produced by the same cognitive or behavioral task under different experimental conditions. In particular, such a study should evaluate the effects of experimental design parameters on the corresponding HGA. We also recommend including a broader range of cognitive and behavioral tasks (e.g., sensory, expressive language, and mental tasks) to expand experimental coverage. From a technological perspective, we suggest translating our results to other established invasive recording techniques such as sEEG.

## 5. Conclusions

In this work, we performed a thorough characterization of HGA in ECoG signals. This characterization showed, for the first time, that the high-gamma frequency band strongly varies across subjects and cognitive and behavioral tasks. We further observed that transients in HGA time courses are band-limited to 10 Hz. The task-related rise time and duration of these HGA time courses depend on the individual subject and the performed cognitive or behavioral task. Interestingly, the task-related HGA amplitudes are comparable across the investigated tasks. All these findings are of high practical relevance, as they provide a systematic basis for optimizing experiment design, acquisition and processing of ECoG signals, and HGA estimation. At the same time, our results reveal previously unknown characteristics of HGA, the physiological principles of which remain to be investigated in further studies.

## Data availability statement

The datasets presented in this article are not readily available because, data-sharing agreements must be approved by both the Institutional Review Boards of Albany Medical College and Asahikawa Medical University. Requests to access the datasets should be directed to JG, gruenwald@gtec.at.

## Ethics statement

The studies involving human participants were reviewed and approved by Institutional Review Boards of Albany Medical College and Asahikawa Medical University. All subjects in this study voluntarily participated in the research experiments, and written informed consent was obtained from each patient before participating in the study.

## Author contributions

JG: conceptualization, formal analysis, and writing—original draft. JG, CK, SS, and PB: methodology and data curation. JG and SS: software, validation, and visualization. JG, CK, and PB: investigation. KK, PB, and CG: resources. JG, CK, SS, and PB: data curation. CK, JS, KK, PB, and CG: writing—review and editing. KK, PB, and CG: supervision and project administration. PB and KK: funding acquisition. All authors contributed to the article and approved the submitted version.
