# Supplementary material for: Characterization of High-Gamma Activity in Electrocorticographic Signals
Source: Front Neurosci. 2023 Aug 7;17:1206120. doi: 10.3389/fnins.2023.1206120 (PMC10440607; doi:10.3389/fnins.2023.1206120)
Supplement: Supplementary file 1 [file Data_Sheet_1.PDF]

# Supplementary Material

## 1 ELECTRODE MONTAGES

Figures S1 to S18 depict the electrode montages for subjects S01–S18 as listed in Table 1 of the main manuscript. Black dots represent those electrodes available for processing, i.e., those electrodes that have not been excluded due to bad signal quality or pathologic brain activity. Purple-shaded areas indicate the electrodes selected for processing a given experimental protocol. In Figures S1 to S11, the indicated and true electrode locations might slightly differ due to the projection onto a standard Montreal Neurological Institute and Hospital (MNI) brain.

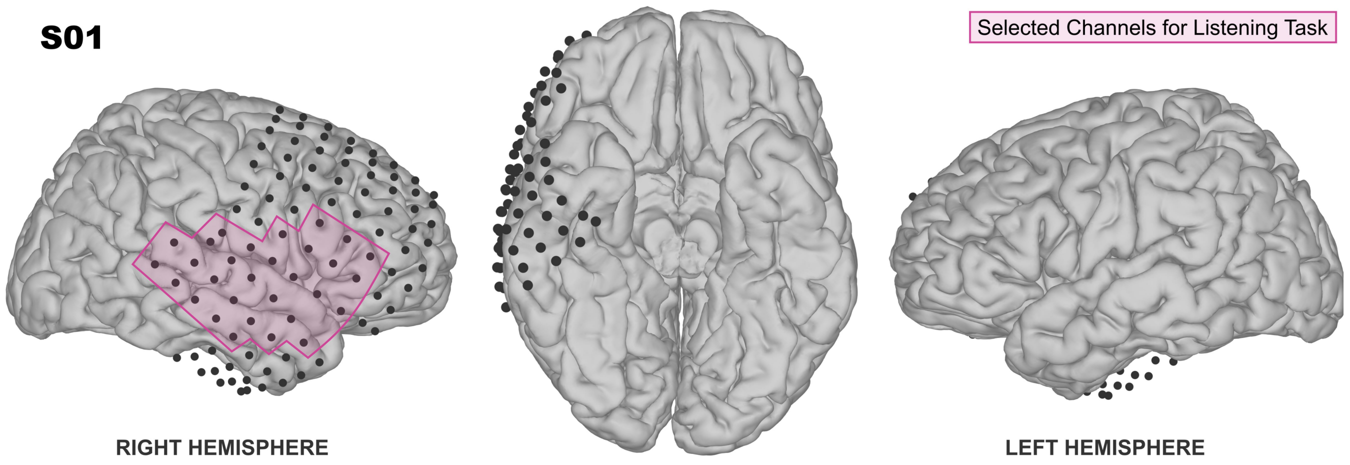


**Figure S1.** Electrode montage of subject S01.

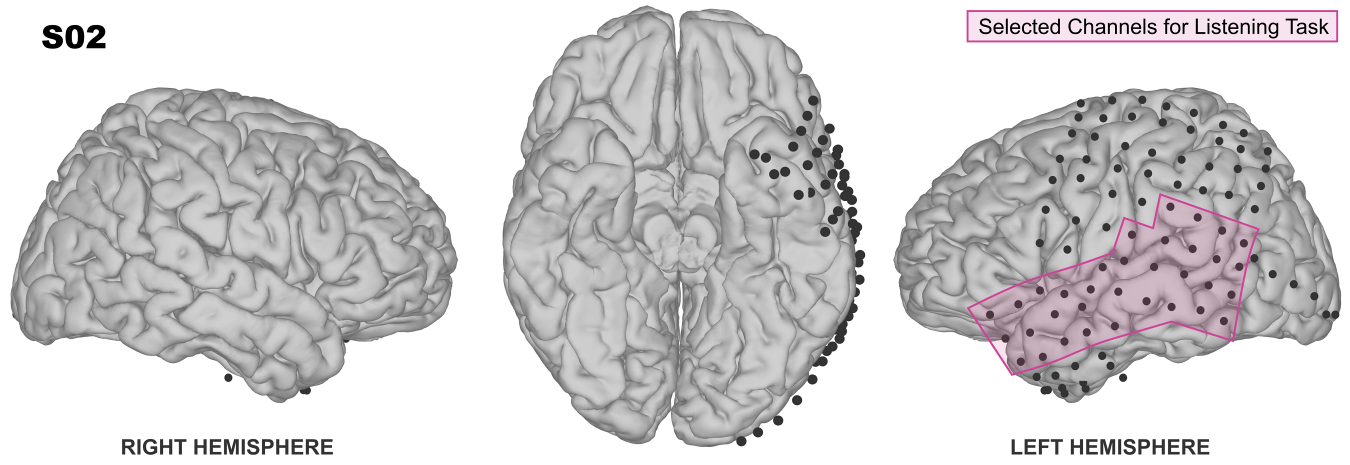


**Figure S2.** Electrode montage of subject S02.

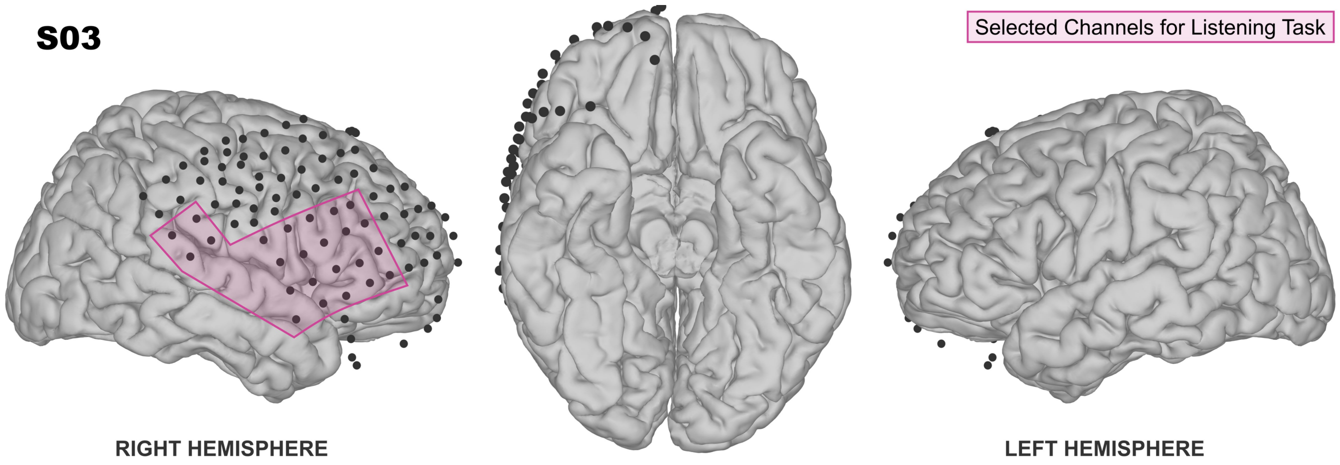


**Figure S3.** Electrode montage of subject S03.

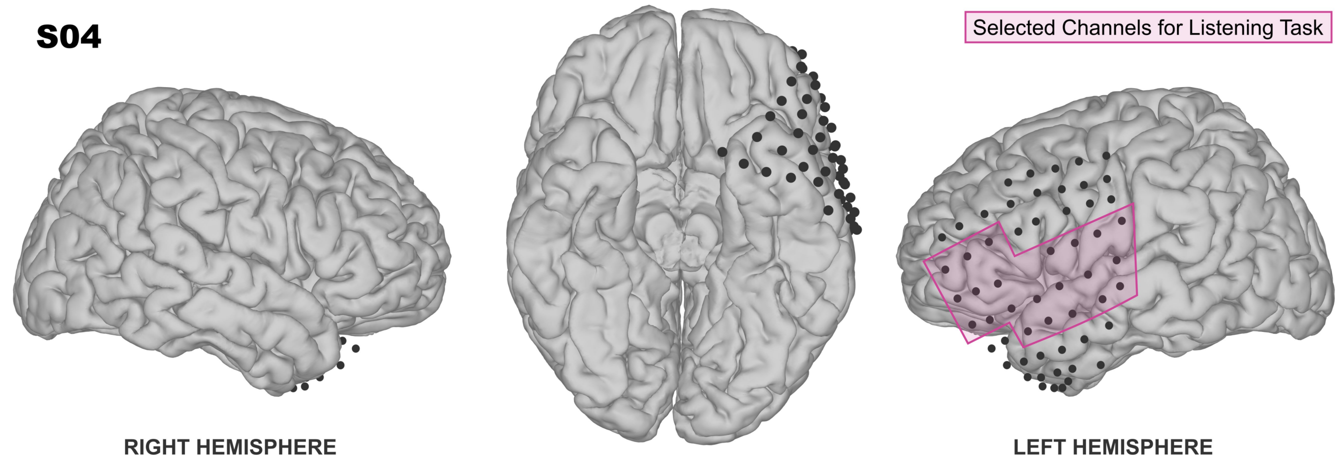


**Figure S4.** Electrode montage of subject S04.

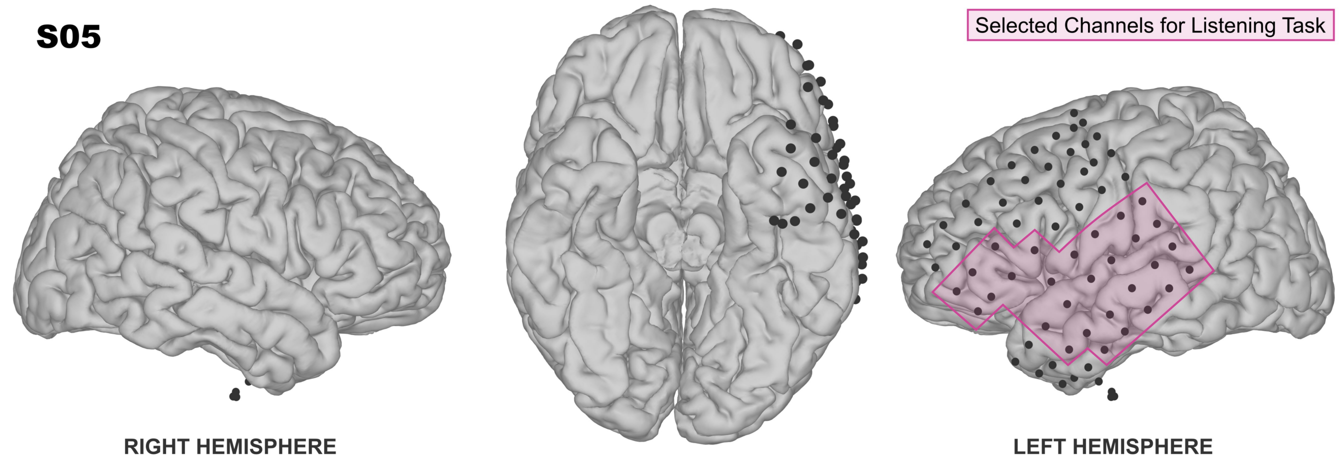


**Figure S5.** Electrode montage of subject S05.

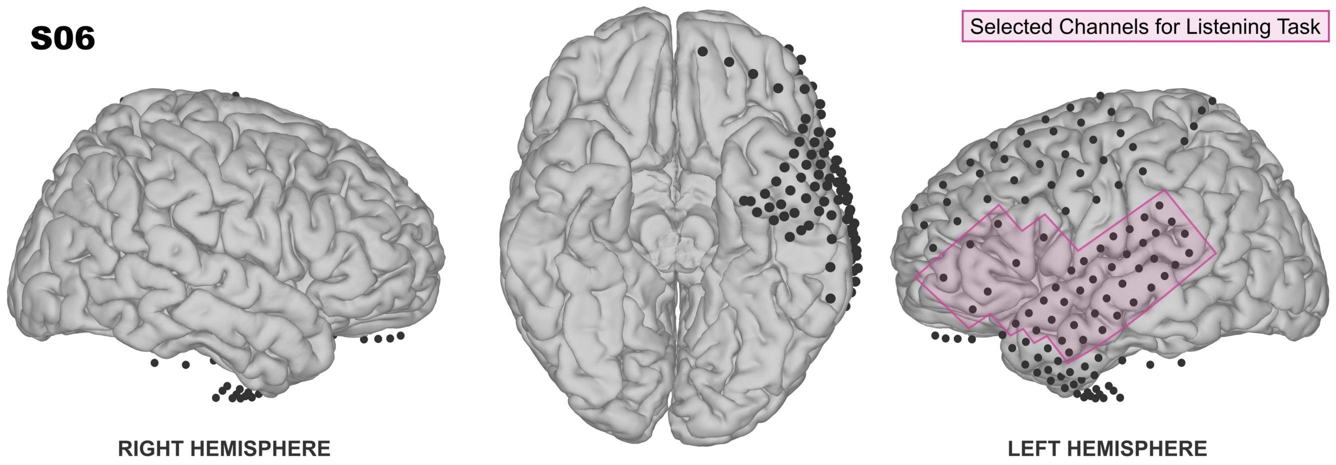


**Figure S6.** Electrode montage of subject S06.

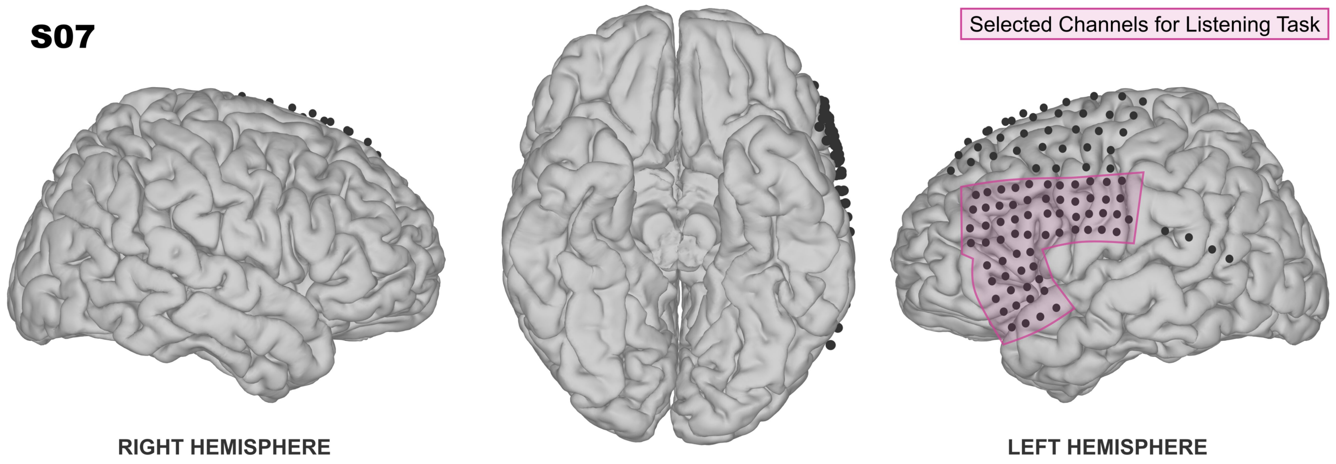


**Figure S7.** Electrode montage of subject S07.

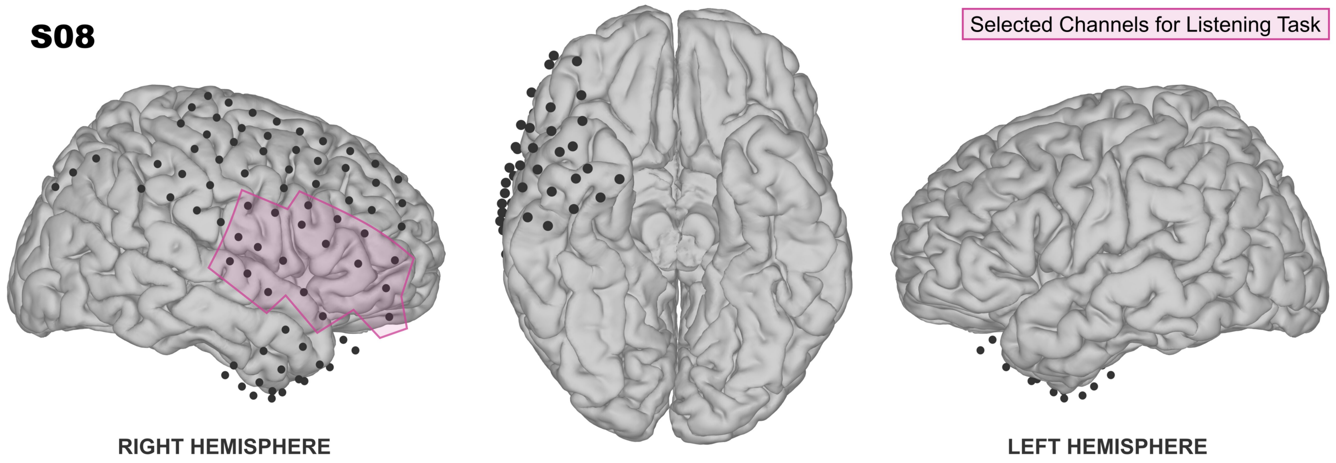


**Figure S8.** Electrode montage of subject S08.

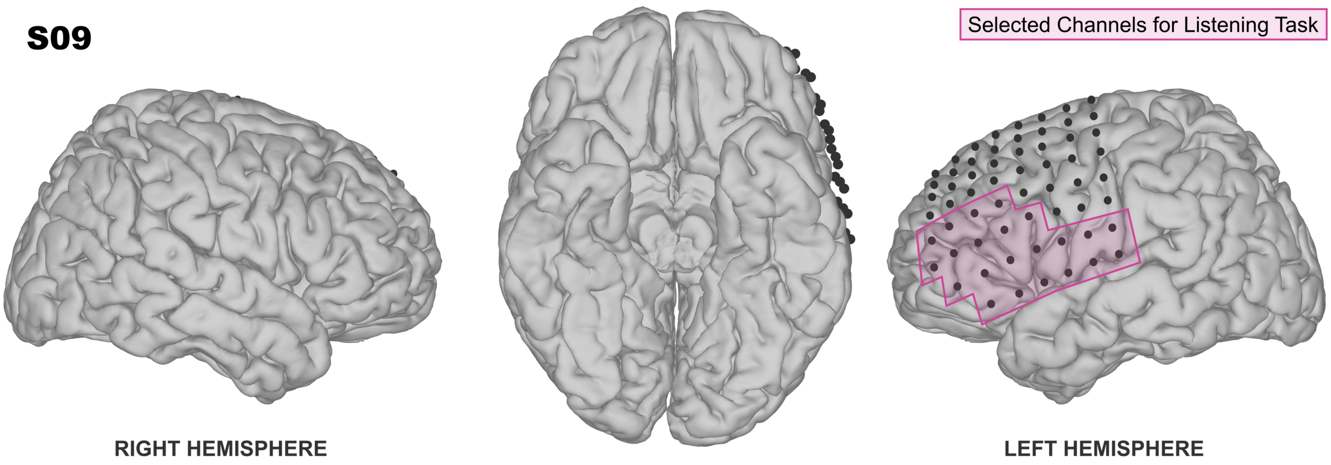


**Figure S9.** Electrode montage of subject S09.

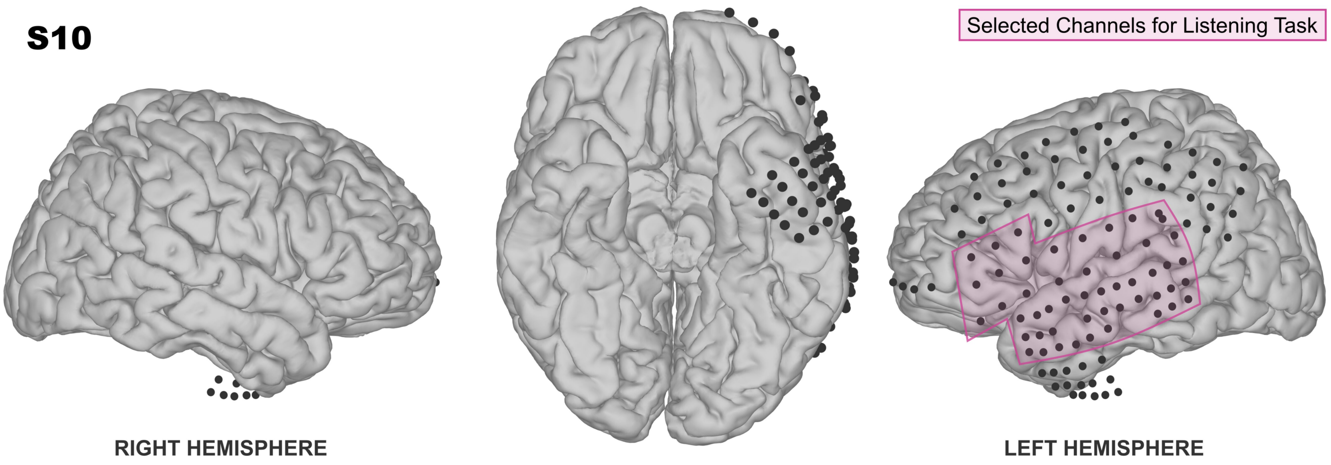


**Figure S10.** Electrode montage of subject S10.

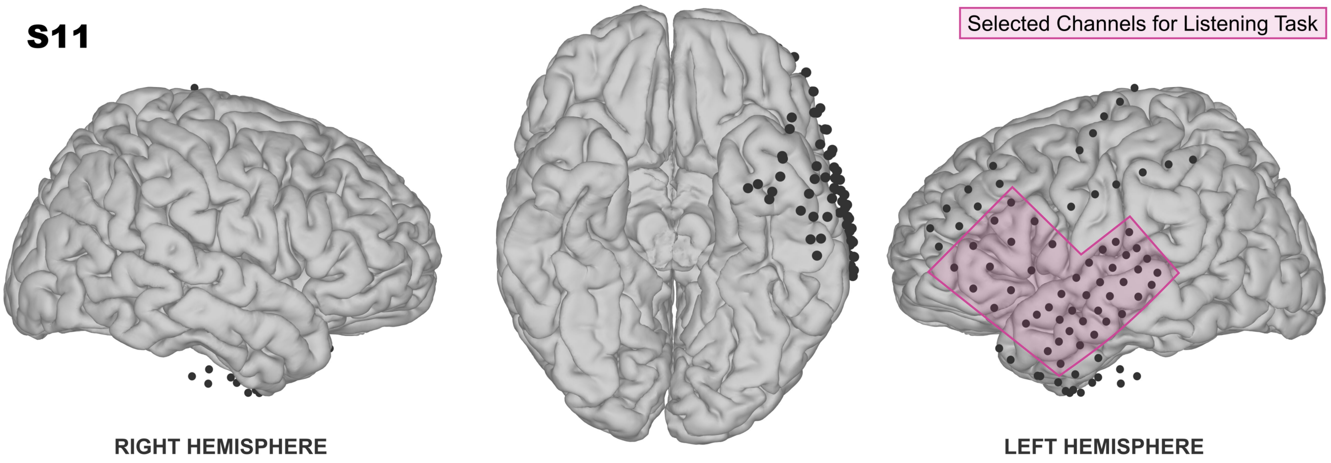


**Figure S11.** Electrode montage of subject S11.

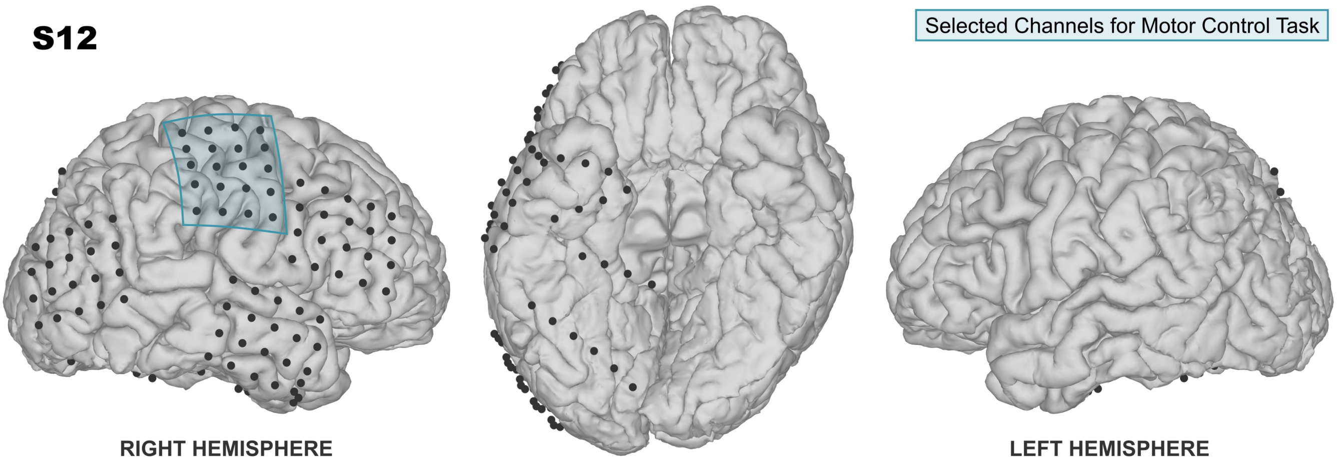


**Figure S12.** Electrode montage of subject S12.

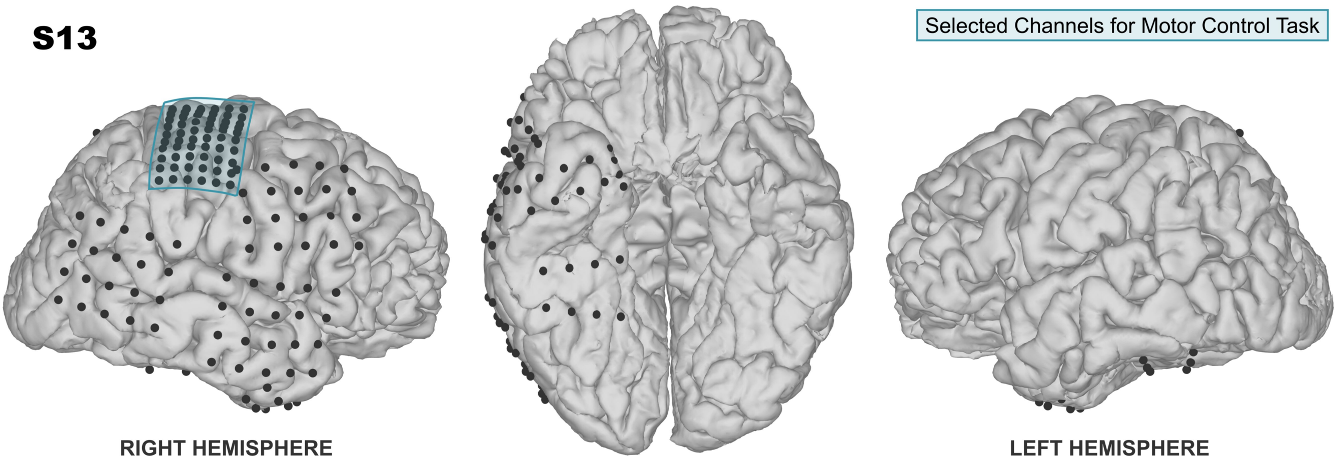


**Figure S13.** Electrode montage of subject S13.

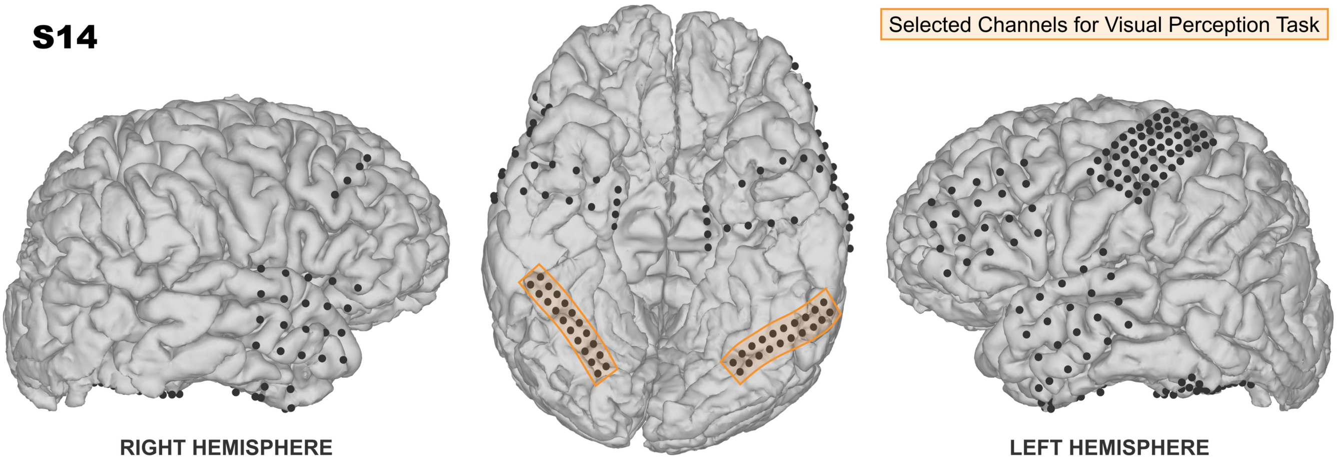


**Figure S14.** Electrode montage of subject S14.

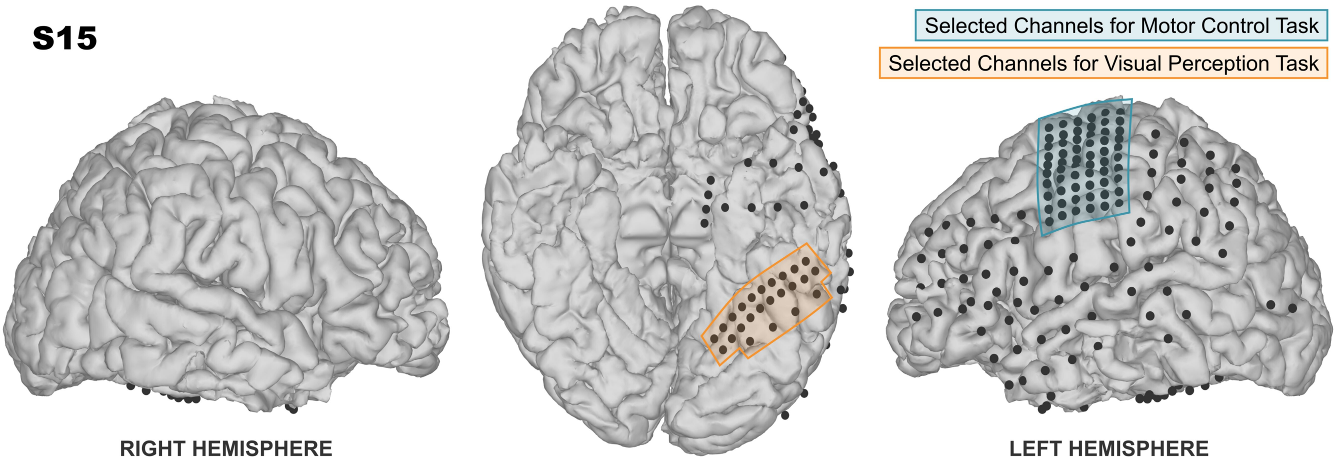


**Figure S15.** Electrode montage of subject S15.

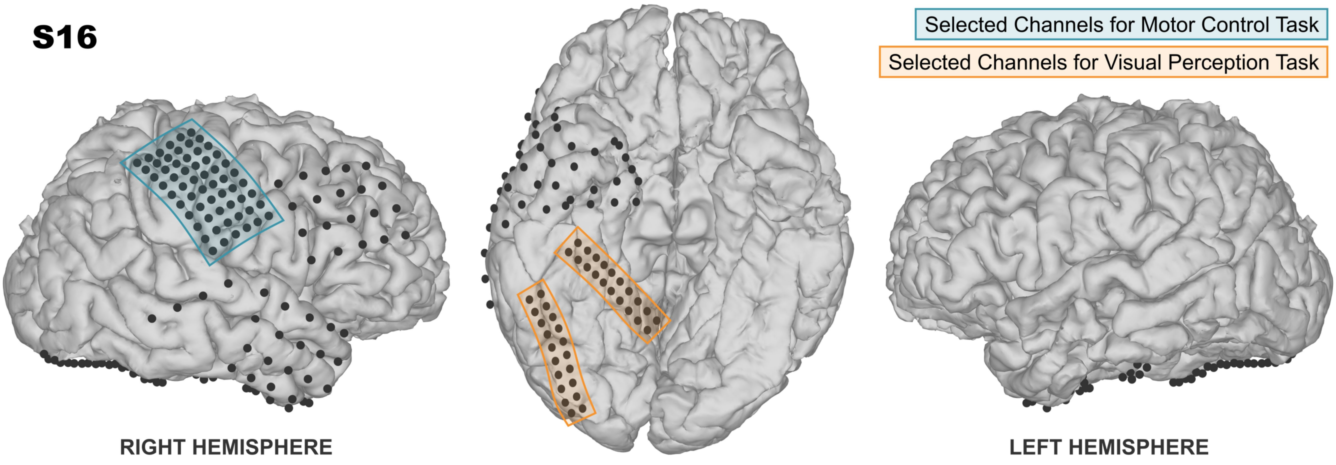


**Figure S16.** Electrode montage of subject S16.

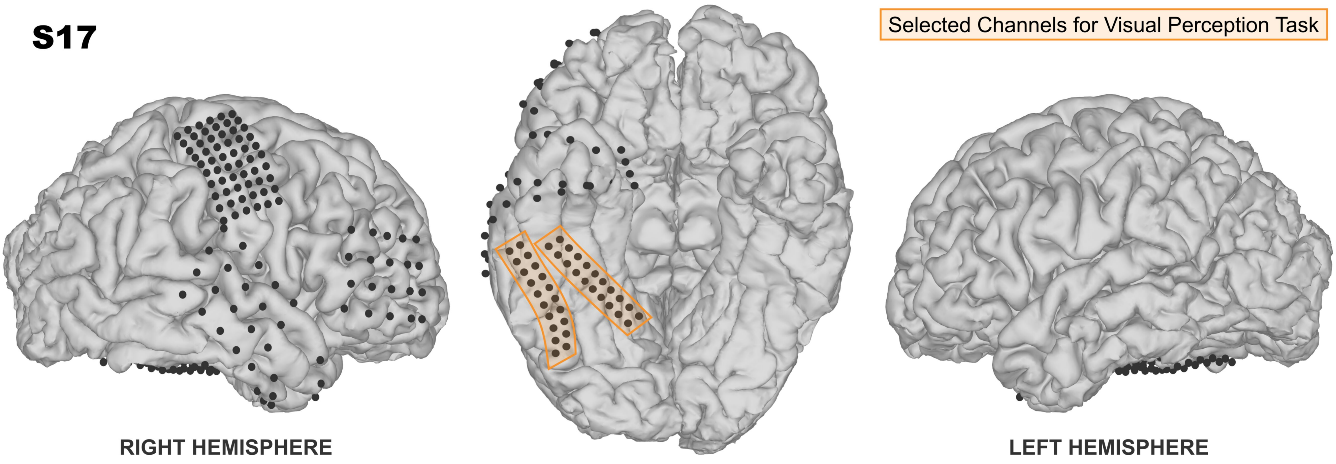


**Figure S17.** Electrode montage of subject S17.

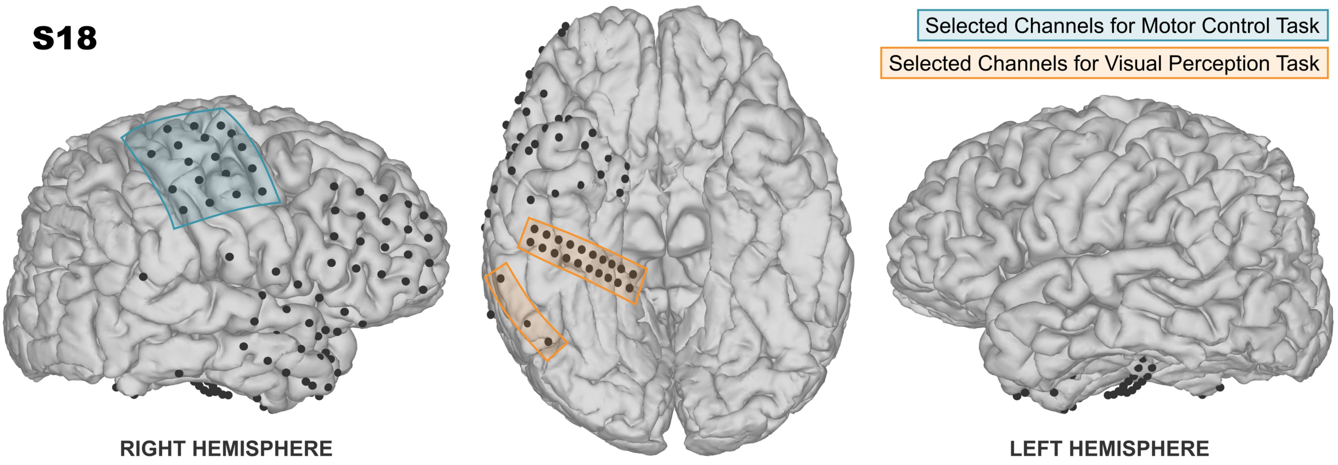


**Figure S18.** Electrode montage of subject S18.

## 2 HGA BASELINE OFFSET CORRECTION

Figure S19 illustrates the baseline offset correction procedure applied in step 3 in Section 2.5.4 in the main manuscript.

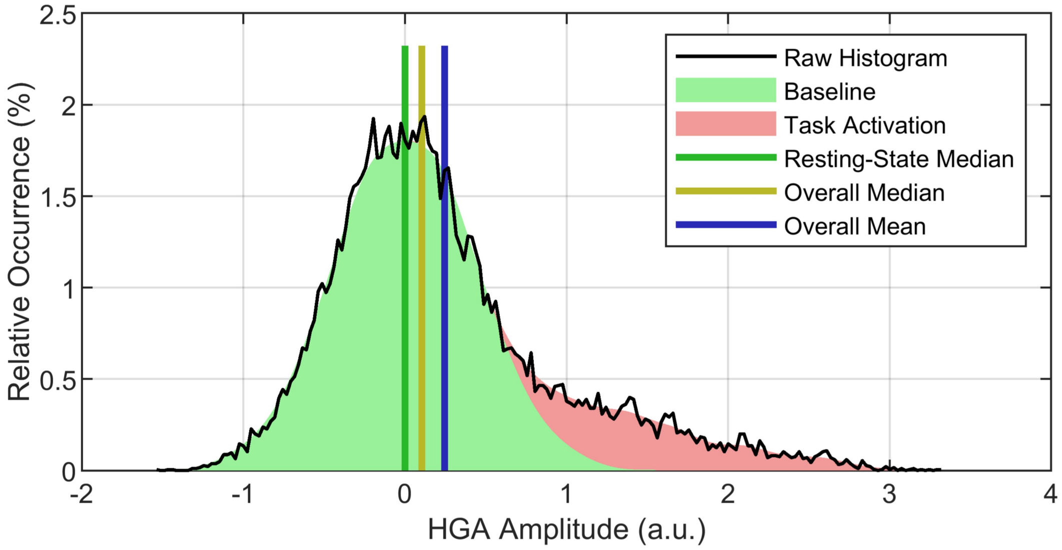


**Figure S19.** Illustration of the baseline offset correction procedure. The raw histogram of HGA estimates can be divided in to a stationary, Gaussian baseline distribution (light green) and a pronounced nonstationary right tail (light red) caused by task-related HGA. Our histogram-based approach described in the main manuscript identifies the resting-state median, which is then used for offset correction. For comparison, we show the median and median based on the overall signals. These metrics are positively biased and therefore inappropriate for baseline correction.

# 3 METHODOLOGICAL CONSISTENCY

To ensure that our results are methodologically consistent, we reproduced all our HGA characterization analyses using the following different methods:

**wBP** (whitened band power) – This is the method described in the main manuscript, Section 2.4.

**rBP** (raw band power) – This method is identical to the wBP method, except that time-domain spectral whitening is disabled.

**HILB** (Hilbert amplitude) – This method is identical to the rBP method, except that the HGA estimates are computed as the envelope of the analytic signal obtained from the Hilbert transform. No log transform is used. For conformity with the other methods, we downsampled the HGA estimates to the target feature rate of 100 Hz.

**logHILB** (log Hilbert power) – This method is identical to the HILB method, except that the signal power is computed as the log-transformed *squared* magnitude of the analytic signal. As a consequence, the logHILB method produces HGA estimates comparable to the rBP method.

## 3.1 High-Gamma Frequency Band

In a first step, we reproduced the high-gamma frequency band analysis described in Section 2.5.2 in the main manuscript. Specifically, we compared HGA estimators using spectral whitening (represented by the wBP method and considered in the main manuscript) and HGA estimators not using spectral whitening (represented by the rBP method).

Figure S20 shows that disabling spectral whitening causes substantial changes to the high-gamma frequency band. Most importantly, the upper cutoff frequency is robustly >300 Hz for all cognitive and behavioral tasks. On the contrary, the lower cutoff frequency is very sensitive, such that the subject consensus is lower compared to methods using spectral whitening. We consider 90–500 Hz, 60–500 Hz, and 80–500 Hz as the high-gamma frequency band for HGA estimators without spectral whitening.

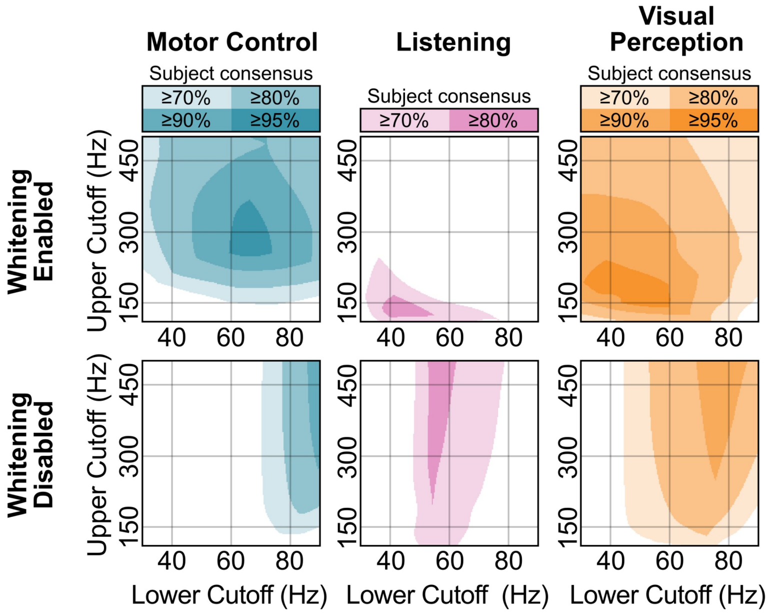


**Figure S20.** Results of the extended high-gamma frequency band analysis. Upper row: Spectral whitening enabled (wBP method, same as in main manuscript). Lower row: Spectral whitening disabled (rBP method).

## 3.2 HGA Bandwidth

In a second step, we reproduced the HGA bandwidth analysis described in Section 2.5.3 in the main manuscript. For this purpose, we recomputed the HGA time courses for the HGA estimators without spectral whitening (rBP, HILB, and logHILB) and without denoising filter. As high-gamma frequency bands, we used the previously obtained 90–500 Hz, 60–500 Hz, and 80–500 Hz for the motor control, listening, and visual perception task, respectively. Figure S21 shows that all four methods yield similar results for the HGA bandwidth, well below 10 Hz in all cases.

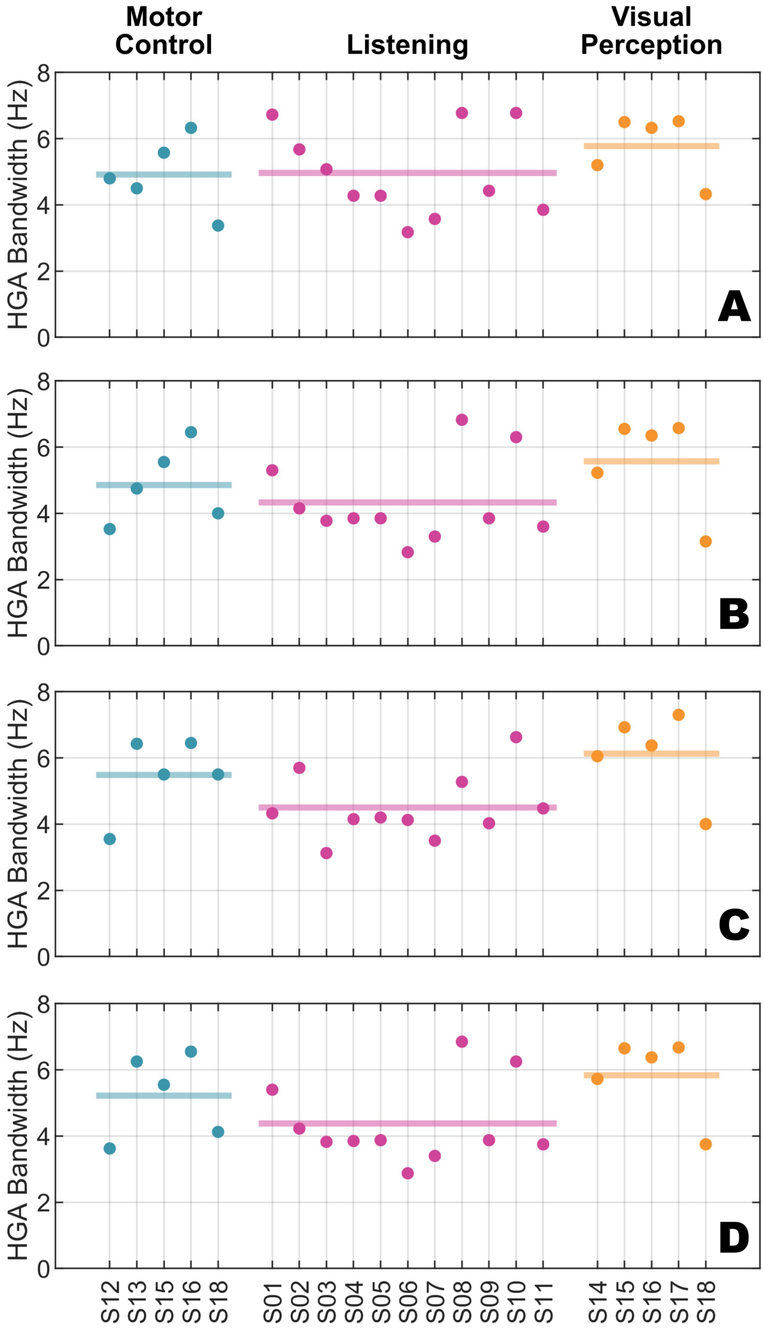


**Figure S21.** Results of the extended HGA bandwidth analysis. Reference wBP method as considered in the main manuscript (A), rBP method (B), HILB method (C), and logHILB method (D).

### 3.3 Temporal Dynamics of HGA

In a third step, we recomputed the temporal dynamics of HGA as described in Section 2.5.4 in the main manuscript. Figure S22 shows the results. Herein, wBP (A) and rBP (B) are almost identical, which confirms that our procedure to extract temporal dynamics is not affected by the use of spectral whitening. In addition, rBP (B) and logHILB (D) show almost identical results, confirming that our procedure is robust whether HGA estimates are based on log band power based or the Hilbert transform. Even rBP (B) and HILB (C) are comparable except for the amplitudes (which can be explained by the absence of the log transform in the HILB method).

Overall, this analysis shows that our procedure to extract the temporal dynamics of HGA is robust to methodological variations.

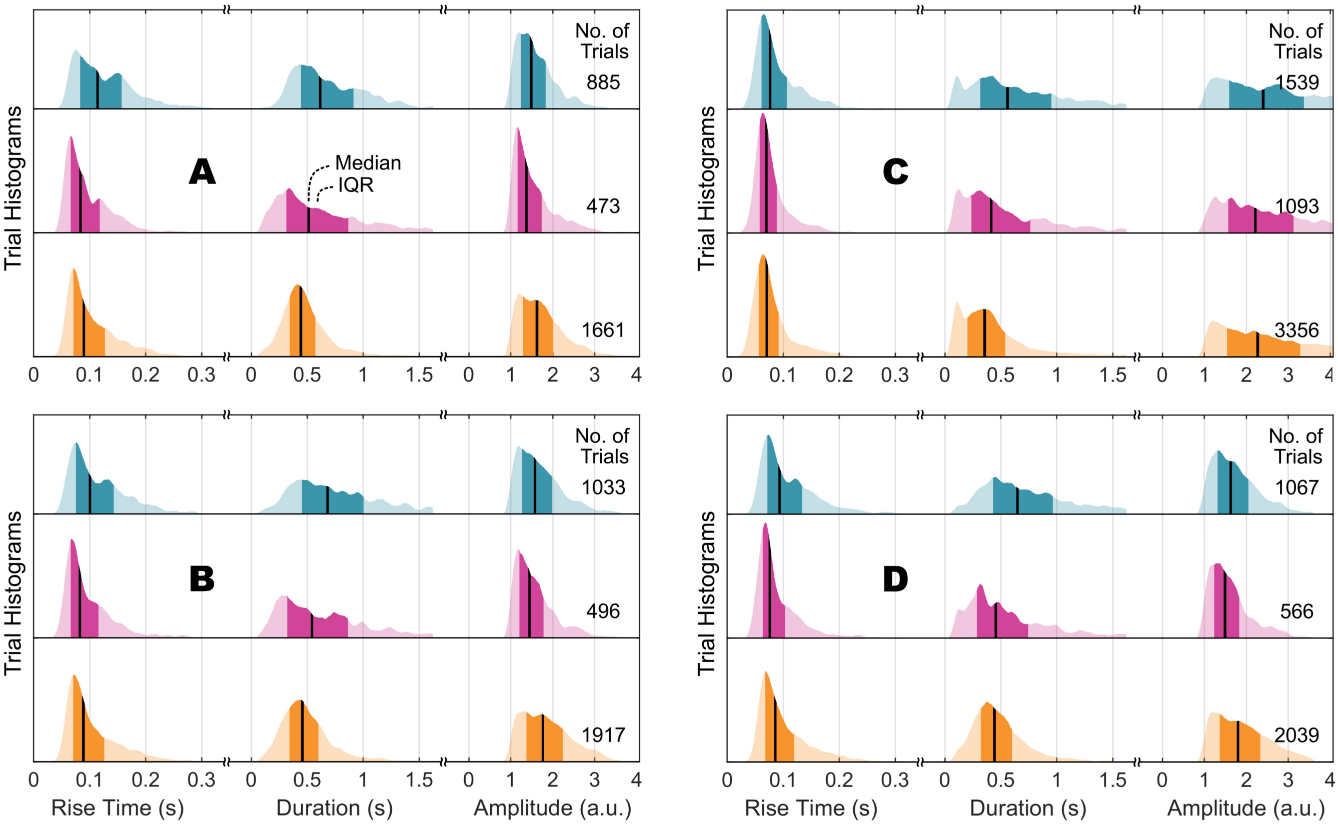


**Figure S22.** Temporal dynamics of HGA; extended results. Reference wBP method as considered in the main manuscript (A), rBP method (B), HILB method (C), and logHILB method (D). For better comparison, we kept axis scaling identical throughout all subplots, which causes occasional clipping of histogram outliers (e.g., amplitudes in (C)).

# 4 HGA TRANSIENTS

This section provides additional analysis of whether HGA may exhibits faster rise times than we were able to detect using our methods described in the main manuscript. This analysis is motivated by the results of Coon and Schalk in 2016, who reported very sharp onset transients in trial-aligned HGA time courses.

## 4.1 Methods

The rationale of this additional analysis is that only the removal of HGA estimation noise can reveal the underlying, physiological HGA time course with all its transients. Unfortunately, standard denoising solutions such as temporal filtering (e.g., lowpass filters) are not suitable because they also smooths potentially fast transients of interest. However, averaging across repeated trials reduces HGA estimation noise without smoothing potentially fast transients. This approach has two requirements: (1) All trial repetitions must be identical, and (2) the individual trials must be identical with respect to their onset.

To satisfy requirement (1), we minimized the trial variations by using data from the visual perception task. As an example, we selected one channel from S15 exhibiting task-related HGA with large amplitudes and fast transients. To satisfy requirement (2), we implemented two onset detection and localization methods described below.

### 4.1.1 Onset Detection and Localization

**Proposed Method.** This procedure uses HGA estimates from the wBP method. In a first step, we applied the denoising filter with a cutoff frequency of 4 Hz. Analogous to step 4 in section 2.5.4 of the main manuscript, we then calculated the symmetric difference of the result to obtain a representation of the signal slope. We then localized the onset of each trial as the maximum slope within a window of 1 second after the trigger. Finally, we aligned the original wBP estimates (i.e., without the denoising filter) around the respective onset and calculated the trial average.

**Coons Method.** For this procedure, we used HILB estimates and applied the original implementation for task-related HGA onset detection published by Coon and Schalk in 2016. We refer to this method as Coons method hereafter.

### 4.1.2 Synthesizing ECoG with Task-Related HGA

To validate onset detection and localization performance, we generated synthesized ECoG signals with realistic, task-related HGA. This involved the following six conceptual steps as illustrated in Figure S23:

(1) We generated an *ECoG base* signal from artificially generated zero-mean white Gaussian noise, where we obtained the characteristic power-law ECoG spectrum through an autoregressive model of order 4. We determined the autoregressive coefficients from real ECoG data and simulated a sampling rate of 1.2 kHz. This ECoG base signal simulates a baseline recording without task-related activity.

(2) To modulate the power of the high-gamma frequency band, we first extracted a *high-gamma base* signal from the ECoG base signal via a bandpass filter (Butterworth order 4, cutoff frequencies 50–200 Hz according to the HGA characterization of the visual perception task).

(3) We subtracted the high-gamma base signal from the ECoG base signal.

(4) We generated an HGA modulation signal to simulate task-related activity during a visual perception task. Building on our experience with HGA in visual perception tasks, we generated this modulation signal with a fast sigmoidal rise and a slow exponential decay. We then developed two scenarios: (a) Short rise time with almost instantaneous onset ($\approx$10 ms) to produce fast transients and high HGA

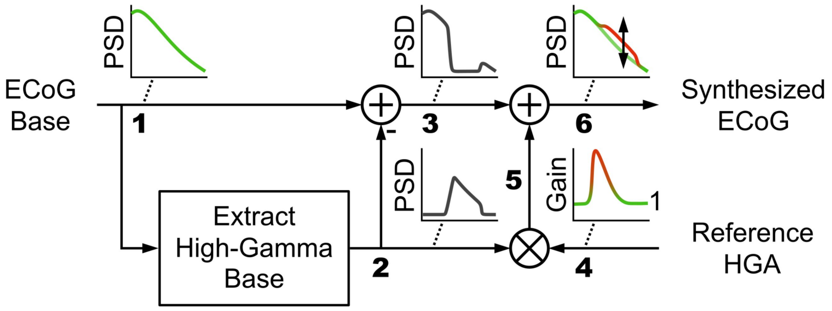


**Figure S23.** Concept of synthesizing ECoG with task-related HGA. The bold numbers refer to the conceptual steps described in the text. For a better reading, the PSD insets' frequency axes and the time axis of the HGA modulation signal inset are not labeled. Green color indicates the resting state, i.e., without task-related activity. Red color indicates task activity.

bandwidth. (b) Long rise time of ≈400 ms to produce slow transients and low HGA bandwidth. With respect to our characterization results (see Figure 4 (G) in the main manuscript), these two scenarios mark the upper and lower ends of task-related rise times (and thus HGA bandwidths) expected in real ECoG recordings. We set the reference onset to 200 ms after the fictitious trigger time point and generated the trials with a random jitter of ±100 ms (uniformly distributed). We refer to this signal as *reference HGA* hereafter.

(5) We element-wise multiplied the high-gamma base signal with the reference HGA signal to obtain high-gamma frequency components with task-related amplitude variation.
(6) We added the result of the previous step to the result of step (3). This yielded the final, synthesized ECoG with task-related HGA.

To validate onset detection and localization performance, we extracted the reference onset time points via thresholding the reference HGA curves at 50% maximum amplitude and compared the resulting jitter before and after trial alignment.

## 4.2 Results

### 4.2.1 Proposed Method

Figure S24 shows the results for the two synthesized ECoG signals (left and middle column) and exemplary real ECoG signals (right column). PSDs of raw HGA estimates (A–C) and trial time courses (D–F) confirm that the synthesized ECoG signals produce realistic task-related HGA. Note that the peaks at 1 Hz and harmonics in the PSD of real HGA (C) correspond to the visual stimulus interval (see Section 2.2.3 in the main manuscript). These peaks are not present in synthesized ECoG (A, B) due to the trial jitter (G, H). The substantial removal of trial jitter in the reference HGA (L, M) compared to the original data (G, H) validates that our onset alignment procedure is working well. This is also qualitatively reflected by real HGA with aligned onset (K) compared to the original data (F).

Overall, this analysis confirms that HGA does not contain faster transients than those obtained from the SNR decomposition method, which uses PSDs of HGA estimates (A–C). Specifically, the smoothed trial averages in (I, J, K) demonstrate that a cutoff frequency of 10 Hz for denoising is sufficient to track all transients in the HGA time course.

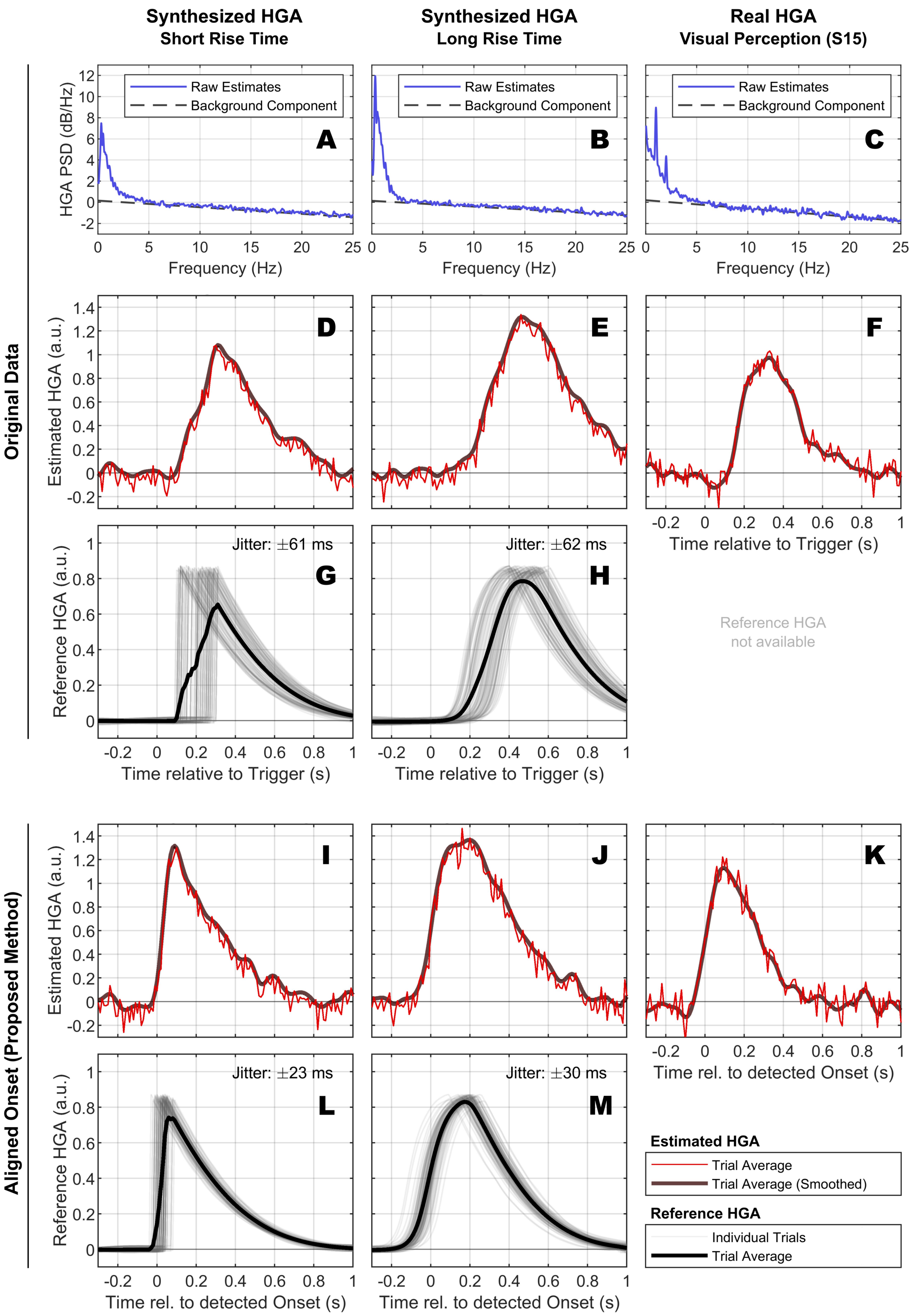


**Figure S24.** Trial averages of HGA estimates. Top: Original data with trial jitter. Bottom: Aligned data using our proposed method. Smoothed HGA estimates were obtained using the denoising filter of the HGA estimator (see Figure 2 in the main manuscript; cutoff frequency 10 Hz). Jitter values are given as standard deviations.

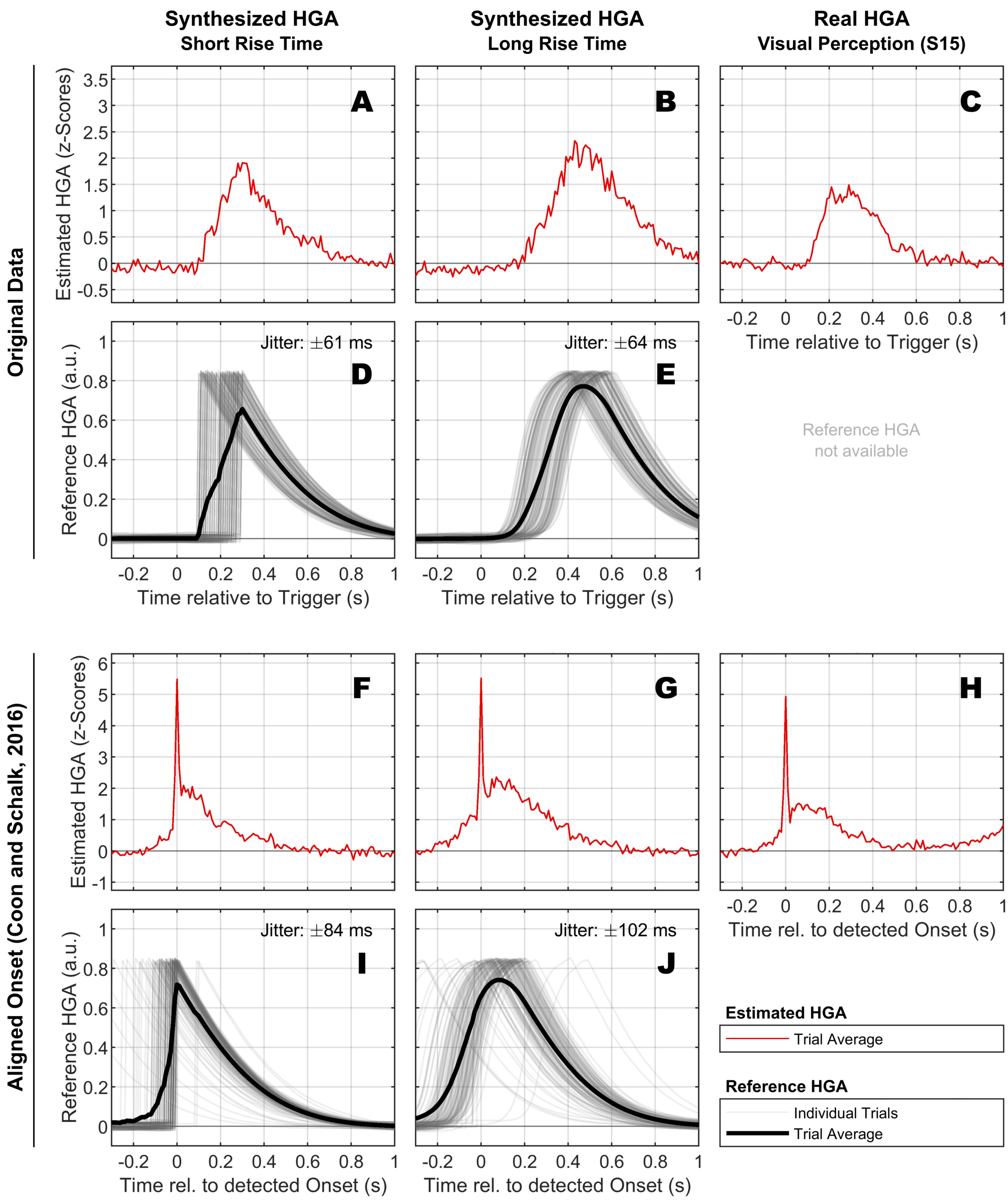


**Figure S25.** Trial averages of HGA estimates. Top: Original data with trial jitter. Bottom: Aligned data using Coons method. Jitter values are given as standard deviations.

### 4.2.2 Coons Method

Figure S25 (F–H) shows that Coons method produces the expected sharp onset peaks. This is surprising for two reasons: (1) The method does not adequately align individual trials because the jitter is not reduced. However, excellent trial alignment would be necessary to obtain such sharp onset peaks. (2) The method produces sharp onset peaks also for the two synthesized HGA datasets. However, it is impossible for this to

reflect any underlying activity, as no such peak is present in the reference HGA. Comparing Figure S25 (H) with Figure S24 (K), it seems also unlikely that such a sharp onset peak is present in real HGA.

Based on these observations, we assume that the observed sharp onset peaks in Figure S25 (F–H) are noise artifacts. This assumption is supported by the fact that Coons method aligns HGA onsets to their *peaks*, which are likely to survive subsequent averaging whether their origin is physiological or not. Nevertheless, Coons method appears to work well on average.

## REFERENCES

Coon, W.G., Schalk, G., 2016. A method to establish the spatiotemporal evolution of task-related cortical activity from electrocorticographic signals in single trials. J Neurosci Methods 271, 76–85. doi:10.1016/j.jneumeth.2016.06.024.
